# QTL mapping for kernel-related traits in a durum wheat x *T*. *dicoccum* segregating population

**DOI:** 10.3389/fpls.2023.1253385

**Published:** 2023-10-02

**Authors:** Ana Paola Valladares García, Francesca Desiderio, Rosanna Simeone, Stefano Ravaglia, Roberto Ciorba, Agostino Fricano, Davide Guerra, Antonio Blanco, Luigi Cattivelli, Elisabetta Mazzucotelli

**Affiliations:** ^1^ Instituto de Conservación y Mejora de la Agrodiversidad Valenciana (COMAV), Universitat Politècnica de València, Valencia, Spain; ^2^ Council for Agricultural Research and Economics (CREA) - Research Centre for Genomics and Bioinformatics, Fiorenzuola d’Arda, Italy; ^3^ Department of Soil, Plant and Food Sciences (DiSSPA), Genetics and Plant Breeding Section, University of Bari Aldo Moro, Bari, Italy; ^4^ S.I.S. Società Italiana Sementi S.p.A, Bologna, Italy; ^5^ Council for Agricultural Research and Economics (CREA) - Research Centre for Olive, Fruit and Citrus Crops, Rome, Italy

**Keywords:** *Triticum dicoccum*, durum wheat, quantitative trait locus, kernel size, kernel shape, kernel weight

## Abstract

Durum wheat breeding relies on grain yield improvement to meet its upcoming demand while coping with climate change. Kernel size and shape are the determinants of thousand kernel weight (TKW), which is a key component of grain yield, and the understanding of the genetic control behind these traits supports the progress in yield potential. The present study aimed to dissect the genetic network responsible for kernel size components (length, width, perimeter, and area) and kernel shape traits (width-to-length ratio and formcoefficient) as well as their relationships with kernel weight, plant height, and heading date in durum wheat. Quantitative Trait Locus (QTL) mapping was performed on a segregating population of 110 recombinant inbred lines, derived from a cross between the domesticated emmer wheat accession MG5323 and the durum wheat *cv*. Latino, evaluated in four different environments. A total of 24 QTLs stable across environments were found and further grouped in nine clusters on chromosomes 2A, 2B, 3A, 3B, 4B, 6B, and 7A. Among them, a QTL cluster on chromosome 4B was associated with kernel size traits and TKW, where the parental MG5323 contributed the favorable alleles, highlighting its potential to improve durum wheat germplasm. The physical positions of the clusters, defined by the projection on the *T. durum* reference genome, overlapped with already known genes (i.e., *BIG GRAIN PROTEIN* 1 on chromosome 4B). These results might provide genome-based guidance for the efficient exploitation of emmer wheat diversity in wheat breeding, possibly through yield-related molecular markers.

## Introduction

Durum wheat [*Triticum turgidum* L. subsp*. durum* (Desf.) Husn., 2n = 4x = 28, AABB] is the most cultivated subspecies of the tetraploid wheats with a global production of 38.1 million tons in 2019 (De Vita and Taranto, 2019; Beres et al., 2020; Xynias et al., 2020). Although durum wheat represents only 5%–8% of the world wheat production, it is the 10th most important crop worldwide, and it is an integral component of the Mediterranean diet due to its use on food as pasta, couscous, and other semolina-based products (Dhanavath and Prasada Rao, 2017; De Vita & Taranto, 2019; Arriagada et al., 2020). Being a mostly rain-fed crop and due to the future climatic scenarios, increasing biomass and thousand kernel weight (TKW) are key goals to develop varieties that could outperform current cultivars under severe climatic conditions and meet rising cereal demand (De Vita and Taranto, 2019; Rahman et al., 2020; Xynias et al., 2020).

Re-introducing genes from wild progenitors or subspecies contributes to enriching the allelic variability of durum wheat germplasm, to cope with current genetic erosion of wheat genepool and to breed more adapted varieties (De Vita and Taranto, 2019; Mazzucotelli et al., 2020; Rahman et al., 2020; Xynias et al., 2020). The potential of cultivated emmer [*Triticum turgidum* subsp*. dicoccum* (Schrank ex Schübl.) Thell.], which is the direct ancestor of wheats, as a genetic source relies in its wider genetic diversity compared with the one of bread and durum wheat, likely due to its long cultivation in a big range of eco-geographical conditions (Zaharieva et al., 2010). The genetic diversity of *T. dicoccum* offers many opportunities for the identification of novel genes/alleles relevant for modern wheat breeding (Zaharieva et al., 2010; Rahman et al., 2020), and many traits have been investigated so far as drought and heat tolerance (Konvalina et al., 2010; Fu et al., 2015; Wang et al., 2019), disease resistance (Piarulli et al., 2012; Desiderio et al., 2014; Olivera and Yue, 2014; Liu et al., 2017; Fatima et al., 2020), insect resistance (Bassi et al., 2019), TKW (Russo et al., 2014; Mangini et al., 2018; Haugrud et al., 2023), and protein content (Dhanavath and Prasada Rao., 2017; Nigro et al., 2019).

Grain yield is a quantitative trait determined by several interrelated plant and grain components. Kernel weight contributes about 20% of the genetic variation in grain yield in bread wheat (Schierenbeck et al., 2021). Moreover, seed morphology descriptors, including kernel size [i.e., length (L), perimeter (P), and area (A)] and shape (roundness), have been demonstrated in determining grain weight and, therefore, grain yield (Cui et al., 2011; Patil et al., 2013; Qu et al., 2021; Mohammadi et al., 2021). In addition, kernel size and shape have a role in the determination of other quality factors for the semolina industry such as test weight, flour yield and milling quality (Tyagi et al., 2015; Wang et al., 2021), and ash distribution (Ficco et al., 2020). The optimum grain morphology ideotype in durum wheat has a large and spherical (thick) shape to maximize endosperm-to-bran ratio, whereas small-sized kernels have the lowest test weight and semolina yield (Ficco et al., 2020). Larger kernels have also shown a positive influence on the seedling vigor and early growth in different crops, as in rice and bread wheat (Avni et al., 2018; Sun et al., 2020).

The understanding of the genetic mechanisms that regulate grain size and shape may facilitate the selection of the ideal kernel architecture through molecular markers (Russo et al., 2014). Most work on QTL mapping for kernel-related traits (size and shape) and kernel weight has been performed in bread wheat [recent reviewed by Brinton and Uauy (2019); Cao et al. (2020), and Gupta et al. (2020)] with around 1,000 QTLs described for kernel related traits (Singh et al., 2022) along with some fine mapping studies [e.g. Zhao et al. (2021)]. Unlike bread wheat, up to date, approximately 300 QTLs have been described in durum wheat for kernel-related traits and located on all 14 tetraploid wheat chromosomes, from the publicly available linkage and association mapping studies until February 2022 [based on 18 studies reviewed by Maccaferri et al. (2019), QTLome; 10 recent studies reviewed by Arriagada et al. (2020); and the works of Desiderio et al. (2019) and Mangini et al. (2021)]. Many detected QTLs are related to TKW, although less information is available on the genetic basis of kernel size and shape (Arriagada et al., 2020). In the cited studies, around 170 loci were found related to TKW, including approximately 90 QTLs related to kernel size factors [L, width (W), P, and A] and 37 QTLs related to kernel shape [W-to-L ratio (WL) and form coefficient (FC)]. Nevertheless, as some of these major QTLs are environment-specific, they should be prudently considered in breeding programs (Arriagada et al., 2020). Indeed, some recent research is based on integrative approaches like meta-analysis of previous QTL studies in order to identify hot spot genomic regions for yield related traits (Arriagada et al., 2020; Yang et al., 2021; Miao et al., 2022). Note that heading date (HD) and plant height (PH) might affect yield and its components, as PH reduction through dwarf major genes increases harvest index and HD delimits grain weight by marking the transition from spike development to grain setting and filling period. Thus, known regulatory genes of phenology and development showed a pleiotropic effect on multiple kernel traits (Arriagada et al., 2020; Mangini et al., 2021; Haugrud et al., 2023).

The knowledge about molecular factors controlling kernel-related traits is mostly extended in rice, with approximately 20 genes already cloned (Chen et al., 2021). The close relationship between wheat and rice has allowed the cloning of orthologous bread wheat genes, whereas such translational approach has not been yet applied in durum wheat. For example, *TaGW2*, the ortholog of *OsGW2*, encoding an E3 ubiquitin ligase involved in the pathway for cell wall expansion, has been demonstrated to control grain weight and kernel architecture in bread wheat (Zhai et al., 2018). Similarly, the bread wheat ortholog of rice gene *BIG GRAIN 1* was mapped on chromosome 4B and proved to be related with auxin transport and regulation of seed growth (Liu et al., 2015; Milner et al., 2021). Most recently, Guo et al. (2022), demonstrated enhanced grain weight and grain yield in wheat upon localized overexpression of the gene *TaCYP78A5*, whose homologous previously shown to affect seed size in several plant species.

The functions of the genes with a role in kernel-related traits are highly diverse (Chen et al., 2021), include the following: i) metabolism of growth regulators such as auxins as, for example, *TaTGW6* (Hu et al., 2016); ii) genes determining cell division and proliferation as *FUWA* (Chen et al., 2015); iii) genes involved in carbohydrate metabolism as *TaSus1* (Mohler et al., 2016); iv) genes coding for proteins involved in ubiquitination processes as *TaSDIR1*-4A (Wang et al., 2020); and v) transcription factors as *TaGL3A* (Yang et al., 2019).

The present study was conceived to unlock and dissect genetic variability behind kernel morphological traits and TKW, by performing a QTL mapping analysis on a recombinant inbred line (RIL) population derived from a cross between a *T. dicoccum* accession and a durum wheat cultivar. The research work included: i) the high-throughput phenotyping of kernels by image analysis, ii) the identification of QTL regions for the related traits and further identification of QTL clusters, iii) a comparison of these QTLs with previous genetic knowledge to highlight stable and novel QTL regions for the mentioned traits, and iv) the identification of candidate genes within the physical positions of the QTL clusters.

## Materials and methods

### Plant materials

**Figure 1:**
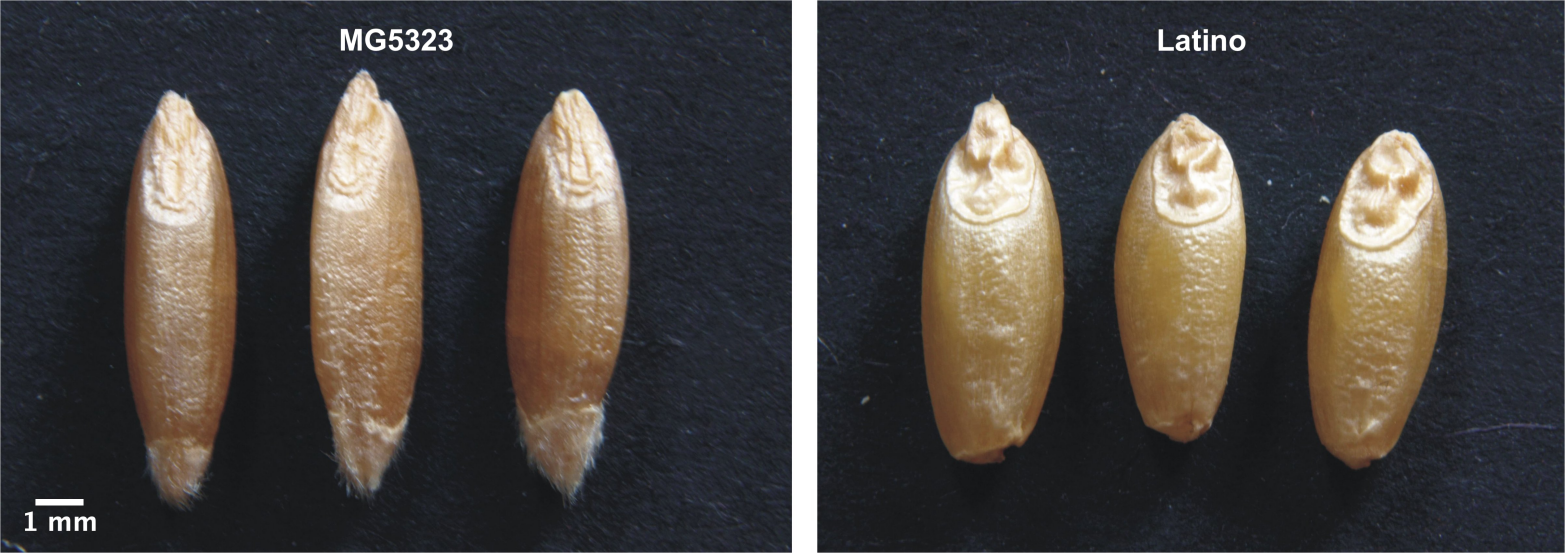
Kernel morphology of parental lines used in this study. Left: *T. turgidum* ssp. *dicoccum* MG5323. Right: *T. turgidum* ssp. *durum cv*. Latino. Reduction scale is reported with a white line representing 1-mm length.

A RIL population of 110 lines developed via single-seed descent of F_2_ plants from a cross between the accession MG5323 of emmer wheat (*T. turgidum* ssp. *dicoccum*), and the durum wheat (*T. turgidum* ssp. *durum*) cultivar (*cv*.) “Latino” was used for the present study. The accession MG5323 (USDA accession number PI 94683) shows longer and thinner kernels than *cv*. Latino (**Figure 1**) and has other important agronomic traits as resistance to powdery mildew, leaf rust and stem rust (Piarulli et al., 2012; Desiderio et al., 2014; Marone et al., 2022), increased protein yield, and high gluten content (De Vita et al., 2006). MG5323 was collected in Armenia and maintained by the National Small Grains Collection (USDA-ARS, Aberdeen, ID, USA). The cultivar Latino (pedigree CAPPELLI/ANHINGA/4/YAKTANA-54//(SEL.14)-NORIN-10/BREVOR/3/ST-64/2*THATCHER) was firstly released by the Federconsorzi (Italy) in 1982 (Desiderio et al., 2014).

### Phenotypic evaluation of the RIL population

The parental lines and the RIL population were evaluated in four different environments (location × year) including Valenzano (BA, Italy) in 2012–2013 (V13), Bologna (BO, Italy) in 2013–2014 (B14), and in Fiorenzuola d’Arda (PC, Italy) in 2014–2015 (F15) and 2019–2020 (F20). A randomized complete block design was developed with two replications for V13 and B14 and three replications for F15 (then reduced to two replications, due to field experimental issues) and F20. Each experimental unit consisted of a single 1-m row with 20–25 plants each. Trials were fertilized following the standard agronomic practices for each location, and weeds were chemically controlled. For further analysis, 110 RILs were considered in each environment, except for F20, where only 103 lines were harvested.

The phenotypic characterization was performed on a random sample of 100 kernels for each experimental unit. Each sample was scanned by Epson Expression 10000XL. Following, the kernel morphology descriptors, presented in **Table 1**, were analyzed by the software WinSEEDLE™ Pro Version 2011a (Regent Instruments Canada Inc.). In addition, for B14, F15, and F20, TKW, PH, and HD were scored for each experimental unit. PH was scored at maturity, and it included spikes. HD was recorded as the number of days between 1 April and the day when 50% of tillers within a plot have the spike emerged from the flag leaf. Three samples of 100 kernels were randomly chosen from the seed bulk of each experimental unit and weighted, and the medium value used to calculate the corresponding TKW was expressed in g/1,000 seeds.

**Table 1:** Kernel morphological traits considered in this study and corresponding definition, ordered from main attributes to derivative attributes, and indicating trait acronym, category, and measure unit [modified from Desiderio et al. (2019)].

Descriptor	Definition	Illustration	Trait category (unit)
Length (L)	The straight distance between the two farthest points on the projected image perimeter.	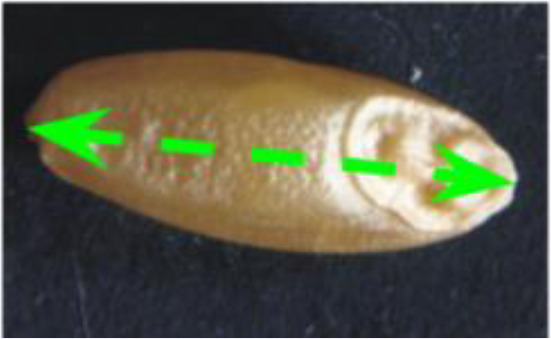	Kernel size (mm)
Width (W)	The maximum width measured perpendicular to length.	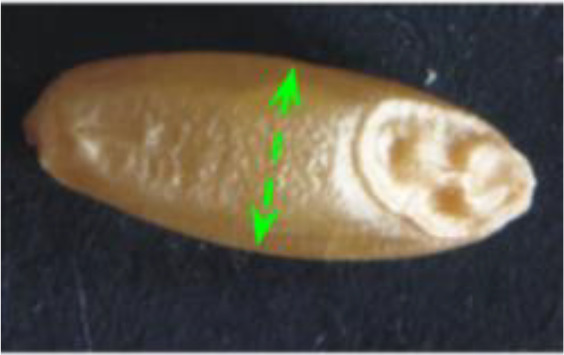	
Perimeter (P)	The length of the seed’s outline.	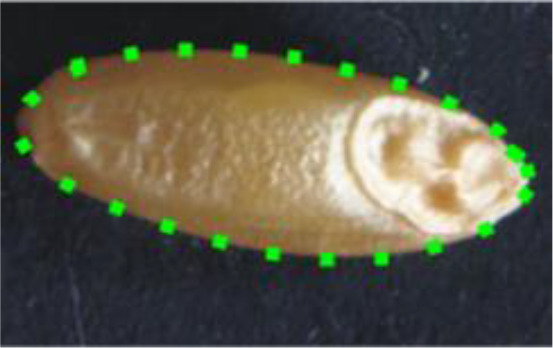	
Area (A)	The two-dimensional area occupied by the seed projection.	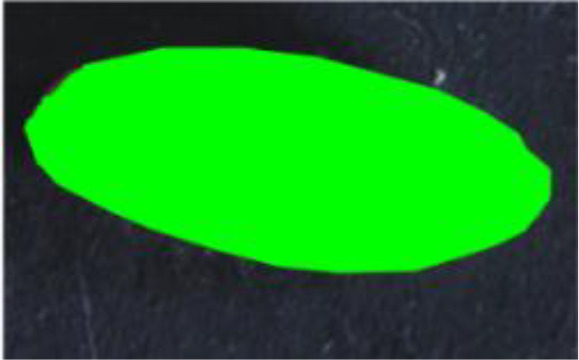	Kernel size (mm^2^)
Width to Length Ratio (WL)	The comparison of the width and length.	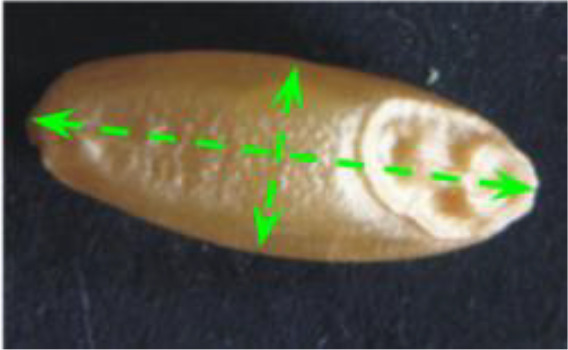	Kernel shape
Form Coefficient (FC)	Indicates the seed shape through the formula 4*π*A/P^2^, where A is area and P is perimeter; with a value of 0 for a filiform object and 1 for a perfect circle.	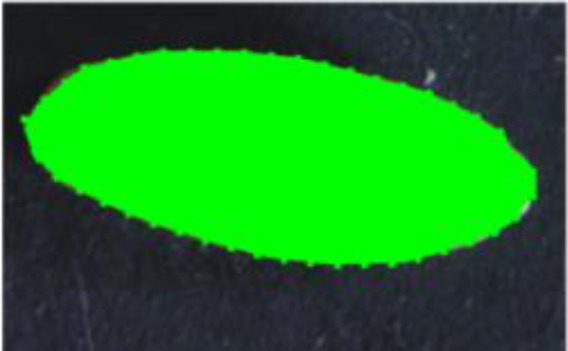	

### Statistical analysis

The statistical analyses were performed in R software (R Core Team, 2021) using the phenotypic data from each environment and for each trait. The Shapiro test and Student’s t-test were performed, using the R/rstatix package (Kassambara, 2021), to evaluate normality of data and the differences between parents, respectively. Descriptive statistical analyses and analysis of variance (one-way ANOVA, *p<* 0.05) were performed to determine the effect of the RILs. Repeatability for each environment was estimated for each trait. Adjusted overall means across environments [best linear unbiased predictions (BLUPs)] were calculated by fitting a linear mixed model through the gamem_met function in R/metan. In this model, environment was considered as fixed effect, genotypes, and genotype × environment interaction (GEI) as random effects. The model was formalized as follows:


$${\text{Y}}_{\text{ijk}}=\text{μ}+\text{Ge}{\text{n}}_{\text{k}}+\text{En}{\text{v}}_{\text{i}}+\text{Re}{\text{p}}_{\text{j}}(\text{En}{\text{v}}_{\text{i}})+\text{Ge}{\text{n}}_{\text{k}}\times \text{En}{\text{v}}_{\text{i}}+{\text{ϵ}}_{\text{ijk}}$$

where $y_{ijk}$ is the response variable (that is the phenotypic value of the trait of interest) measured in for the genotype *k*, in the environment *i* and replicate *j*; μ is the overall mean; *Env* is the effect of the environment *i*; *Rep* is the effect of replicate *j* on each environment *i*; *Gen* is the effect of the genotype *k; Gen* x *Env* is the interaction effect between genotype *k* and environment *i*; and ϵ is the error associated with each phenotypic value of the response variable $y_{ijk}$ . This model assumes that the random effects of *Gen* follow a normal distribution with mean 0 and variance $\sigma_{g}^{2}$ . Likewise, the model assumes $\epsilon ijk∼(0,\sigma_{\epsilon }^{2})$ , that is the error terms ε are independent and normally distributed with mean 0 and variance $\sigma_{\epsilon }^{2}$ . The across-environment broad-sense heritability (H^2^) was estimated from the BLUP model through the following formula:


H2= (σg2)(σg2+σi2+σr2)

where $\sigma_{g}^{2}$ is the genotypic variance, $\sigma_{i}^{2}$ is the GEI variance, and $\sigma_{r}^{2}\;$ is the residual variance. Pearson’s correlation coefficients were calculated for all trait combinations based both on the data recorded for each environment and for BLUPs. All these analyses were performed by using R/metan package (Olivoto and Lúcio, 2020).

### Whole-genome sequencing of MG5323 and SNP calling

Genomic DNA of MG5323 was isolated and purified with the NUCLEO SPIN PLANT II (Macherey-Nagel) following the manufacturer’s instructions. Illumina paired-end [2 X 150 base pairs (bp)] whole-genome sequencing of MG5323 was obtained by Illumina NovaSeq 6000^®^ at Biodiversa About 300 Gb of raw reads (25X coverage of the 12 Gb durum wheat genome) were subjected to quality control using FastQC version 0.11.7 (Andrews, 2010). Subsequently, reads containing adapter sequences were discarded using cutadapt version 1.17 (Martin, 2011), and the resulting FASTQ files were trimmed to a base quality of 10 from both ends with TRIMMOMATIC version 0.30 (Bolger et al., 2014) using the following parameters: LEADING = 10, TRAILING = 10, SLIDINGWINDOW = 4, and MINLEN = 50. Filtered reads were aligned to the reference sequence of *T. turgidum* L. ssp. *durum, cv.* Svevo (Maccaferri et al., 2019) using Burrow-Wheeler Aligner (BWA-MEM) version 0.7.15 with default parameters (Li and Durbin, 2009), whereas duplicated reads were marked in the alignment file using the “MarkDuplicates” command of Picard software (http://broadinstitute.github.io/picard).

Genetic variants were called from the resulting alignments with marked duplicated reads using SAMtools/BCFtools pipeline version 1.7 with BCFtools call parameter set to -m and -v. Beyond SAMtools/BCFtools pipeline, FreeBayes version 1.0.0 (Garrison and Marth, 2012) was additionally used for Single Nucleotide Polymorphism (SNP) calling using default parameters. SNP calls detected using both SAMtools/BCFtools pipeline and FreeBayes were subsequently filtered for including variants supported with more than 20 reads and a mapping quality higher than 50. The subset of filtered SNPs called with both SAMtools/BCFtools pipeline and FreeBayes was considered for further analyses. Along with SNPs, the mentioned pipeline identified raw small indels, which were hard filtered using the same parameters used for other genetic variants. The annotation and prediction of functional effect of the genetic variants have been done using the SnpEff toolbox version 4.3t (Cingolani et al., 2012) and the Svevo genome annotations (Maccaferri et al., 2019) including low-confidence and high-confidence annotated genomic features.

### QTL mapping

The high-density genetic map used in this study was previously constructed (Desiderio et al., 2014; Maccaferri et al., 2015) with a total of 10,840 markers assembled into 14 linkage groups corresponding to the 14 durum wheat chromosomes and an overall map length of 2,363.4 cM. For each trait, the R/qtl package (Broman et al., 2003) was used for QTL analysis with the mean values for each genotype in each single environment and the BLUP values as adjusted mean values for the combined data. The procedure described by Desiderio et al. (2019) was performed as follows: (i) a permutation test to define the logarithm of odds (LOD) significance level with a genome-wide significance level of 5% after 1,000 permutations; (ii) initial scan of the genome using the simple interval mapping (SIM) with a 1-cM step; (iii) evaluation of the position and effect of the QTLs with the multiple imputation method [composite interval mapping (CIM)]; and (iv) the “addqtl” command to search for additional QTLs. When more QTLs were identified for the trait under consideration, a model containing the QTLs and their possible interactions were tested by the “addint” command. If these putative loci remained significant, then the “refineqtl” command re-evaluated the QTL positions based on the full model.

The additive effects of QTLs were estimated as half the difference between the phenotypic values of the respective homozygotes. If a QTL was found close to the threshold, estimated by permutation, and co-located with a significant QTL, then it was considered as putative QTL. The confidence intervals (CIs) of each QTL were determined as proposed by Darvasi and Soller (1997). Next, for each trait, QTLs found in more than one environment were considered to correspond to the same stable QTL, provided that CIs were overlapping and that the additive effect was conferred by the same parent. Furthermore, QTLs were named according to the rule “Q + trait code + chromosome.locus number,” where Q stands for QTL, trait code refers to the trait acronym presented in **Table 1**, and the last refers to the wheat chromosome on which the corresponding QTL is located. If two QTL are on the same chromosome, then a consecutive number (“.1, .2, .3”) was added.

### Clustering of QTL and identification of candidate genes

To compare QTLs identified in the present study with data from literature and hypothesize candidate genes, the durum wheat reference genome was used as framework to combine physical and genetic information. This comparison procedure included two steps to project current knowledge on the durum wheat reference genome: (i) updating the tetraploid QTLome provided by Maccaferri et al. (2019), with the most recent QTLs for the kernel-related traits retrieved by a literature survey from the publicly available linkage and association mapping studies until August 2022 (Desiderio et al., 2019; Arriagada et al., 2020 and Mangini et al., 2021; **Supplementary Material, Appendix A**); and (ii) compiling a list of wheat and/or rice cloned genes with known functions affecting kernel size, shape, and kernel weight; their sequences were used as a query to perform a BLAST against the durum wheat reference genome to define their genomic positions [**Supplementary Material, Appendix B**; updated from Desiderio et al. (2019) until 1 February 2022].

Other steps were necessary thereafter to anchor the best results of the present study on the reference genome: (i) grouping the QTLs identified in the present study by defining QTL clusters as regions where QTLs for different traits co-located in the Latino x MG5323 map (based on total or partial overlapping of CIs) and defining the QTL with highest LOD and R^2^ values within each cluster as the major QTL of that cluster; (ii) initial anchoring of peak and flanking SNP markers of the best QTL of each cluster on the tetraploid wheat consensus map and defining the related genetic position and selecting the coinciding/nearest QTL from previous studies; (iii) for each cluster, projecting the CI on the durum wheat reference genome assembly (Svevo.v1, Maccaferri et al., 2019) by BLASTing nucleotide sequence of CI flanking markers on Svevo.v1 at https://plants.ensemble.org, upon an intermediate step onto the consensus map of the tetraploid wheat (Maccaferri et al., 2015) in order to use more and reliable markers as bridge and thus increase the consistency and accuracy for the genome projection; (iv) hypothesizing candidate genes within the physical interval of the QTL clusters by screening high-confidence Svevo genes based on their functional annotation (previously obtained via blast2GO PRO, available at https://figshare.com/s/2629b4b8166217890971); (v) in addition, based on the assumption that *cvs*. Latino and Svevo have highly similar (0.85) genome sequence similarity (Mazzucotelli et al., 2020; unpublished exome data), identifying polymorphisms between the MG5323 genome and the Svevo v1 assembly (Maccaferri et al., 2019) in promoter and gene sequences of candidate genes and evaluating their possible effects based on SnpEff (Cingolani et al., 2012); and (vi) analyzing the bread wheat homologous of candidate genes for their transcriptional profile in different tissues/organs (leaf, grain, root, and spike) and at different developing stages (seedling, vegetative, and reproductive). B wheat (*cv.* Chinese Spring) homologous on the IWGSC RefSeqv1.1 genome assembly was retrieved from the Triticeae Gene Tribe homology database (http://wheat.cau.edu.cn/TGT/) (Chen et al., 2020), whereas gene expression data were downloaded from the ExpVIP platform (Wheat Expression Browser, www.wheat-expression.com; Ramirez-Gonzales et al., 2018), which collects published transcriptome data on bread wheat. Transcript abundances were expressed in log2 (transcript per million).

## Results

### Phenotypic characterization of the RIL population

The two parents and the RILs were evaluated for traits related to kernel morphology, size, and weight, and HD and PH in four environments and BLUP across the four environments were calculated (**Table 2**). This analysis shows significant differences (*p*< 0.01) between the two parental lines in each environment and across them for most of the traits, except for A that was only statistically significant in B14. As expected, MG5323 obtained greater values for L and P (kernels longer and narrower), PH and HD and a lower value of kernel weight were compared with that in the cv. Latino. Furthermore, about the RIL mean values in the different environments, the L ranged from 7.9 mm to 8.3 mm, the W ranged from 3.0 mm to 3.2 mm, the P ranged from 18.8 mm to 19.6 mm, the A ranged from 18.6 mm^2^ to 20.3 mm^2^, WL ranged from 0.38 to 0.41, FC ranged from 0.65 to 0.67, TKW ranged from 44.3 to 53.5 g, HD ranged from 30 to 45 days, and PH ranged from 100.8 cm to 106.9 cm. Broad-sense heritability (H^2^) values calculated for each trait in each environment and on BLUPs were high (0.80–0.99), the highest values obtained by L and kernel shape traits (FC and WL).

**Table 2:** Summary of the phenotype data for the nine traits analyzed in the parents and in the MG5323 x Latino RIL mapping population.

Trait	Environment	Parents			RIL						
		Latino	MG5323	*p*-value	Min	Max	Range	Mean	Repeatability	SD	CV%
L	V13	7.47	8.64	***	6.693	9.43	2.737	7.908	0.9	0.449	5.7
	B14	7.66	8.61	****	6.782	9.422	2.64	8.167	0.97	0.486	6
	F15	7.75	9.12	**	6.863	9.939	3.076	8.275	0.97	0.502	6.1
	F20	7.6	9.19	***	6.892	9.524	2.632	8.061	0.99	0.459	5.7
	BLUP	7.64	8.9	–	6.95	9.385	2.434	8.117	\	0.418	5.2
W	V13	3.32	2.92	*	2.708	3.725	1.018	3.158	0.83	0.176	5.6
	B14	3.24	2.72	****	2.218	3.66	1.443	3.06	0.92	0.22	7.2
	F15	3.42	2.96	**	2.698	3.807	1.109	3.245	0.92	0.189	5.8
	F20	3.28	2.82	***	2.364	3.468	1.105	3.016	0.98	0.176	5.8
	BLUP	3.32	2.87	–	2.818	3.451	0.634	3.124	\	0.121	3.9
P	V13	18.19	20.1	**	16.465	21.773	5.308	18.792	0.87	0.919	4.9
	B14	18.28	19.69	****	15.651	22.057	6.406	19.059	0.96	1.005	5.3
	F15	18.75	21.07	**	16.576	23.253	6.677	19.569	0.96	1.02	5.2
	F20	18.34	20.91	***	16.503	21.994	5.491	18.941	0.98	0.932	4.9
	BLUP	18.43	20.48	–	16.748	21.584	4.836	19.127	\	0.819	4.3
A	V13	19.53	19.04	ns	14.876	24.336	9.46	19.139	0.83	1.443	7.6
	B14	18.73	17.36	****	11.581	22.778	11.196	18.973	0.92	1.791	9.5
	F15	20.34	20.35	ns	14.71	25.621	10.911	20.332	0.94	1.742	8.6
	F20	19.19	19.48	ns	14.023	22.692	8.669	18.564	0.97	1.44	7.8
	BLUP	19.37	19.35	–	15.321	22.132	6.811	19.305	\	1.121	5.8
WL	V13	0.45	0.34	***	0.322	0.518	0.196	0.407	0.94	0.03	7.4
	B14	0.42	0.32	****	0.29	0.527	0.237	0.376	0.96	0.034	9
	F15	0.44	0.33	***	0.321	0.519	0.198	0.394	0.95	0.032	8.1
	F20	0.43	0.31	****	0.304	0.501	0.197	0.376	0.99	0.033	8.7
	BLUP	0.44	0.32	–	0.329	0.505	0.176	0.388	\	0.027	7
FC	V13	0.73	0.59	***	0.566	0.792	0.225	0.671	0.94	0.036	5.4
	B14	0.7	0.56	****	0.514	0.803	0.289	0.656	0.94	0.039	6
	F15	0.73	0.58	***	0.573	0.804	0.231	0.668	0.95	0.037	5.6
	F20	0.72	0.56	***	0.543	0.785	0.242	0.651	0.99	0.04	6.1
	BLUP	0.72	0.57	–	0.583	0.781	0.198	0.661	\	0.031	4.7
TKW	B14	51.02	37.38	****	20.167	62.9	42.733	48.009	0.9	6.977	14.6
	F15	59.68	50.43	ns	29.367	68.667	39.3	53.516	0.9	6.64	12.4
	F20	53.53	44.8	**	24.3	56	31.7	44.31	0.96	5.435	12.3
	BLUP	54.99	45.22	–	35.52	58.524	23.005	48.794	0.8	3.963	8.2
HD	B14	20	40	*	13	44	31	30.491	0.94	6.391	21
	F15	34	44.33	****	19	51	32	38.306	0.87	3.777	9.9
	F20	38.67	52.67	**	34	61	27	45.503	0.95	4.829	10.6
	BLUP	33.52	47.69	–	28.974	48.213	19.239	39.051	\	3.664	9.4
PH	V13	83.83	128	**	62.666	141.666	79	106.921	0.97	21.649	20.3
	B14	89	120	*	62.667	138.333	75.666	102.954	0.89	17.513	17.1
	F15	72.8	125.8	***	62.333	138.333	76	100.76	0.98	20.091	20
	BLUP	81.92	125.51	–	65.995	130.28	64.285	103.516	\	18.016	17.5

Significance is denoted as ****p< 0.0001, ***p< 0.001, **p< 0.01, and *p< 0.05 between parental lines based on Student’s t-test; ns, not significant; (-), not available; RIL, recombinant inbred lines; BLUP, best linear unbiased prediction; SD, population standard deviation; CV, variation coefficient.

Traits are denoted as L, length; W, width; P, perimeter; A, area; WL, width-to-length ratio; FC, form coefficient; TKW, thousand kernel weight; HD, heading days; and PH, plant height. Environment acronyms are as follows: V13, Valenzano 2012–2013; B14, Bologna 2013–2014; F15, Fiorenzuola d’Arda 2014–2015; and F20, Fiorenzuola d’Arda 2019–2020. For more details on trait and environment description, please refer to **Table 1** and the Materials and Methods section.

**Figure 2:**
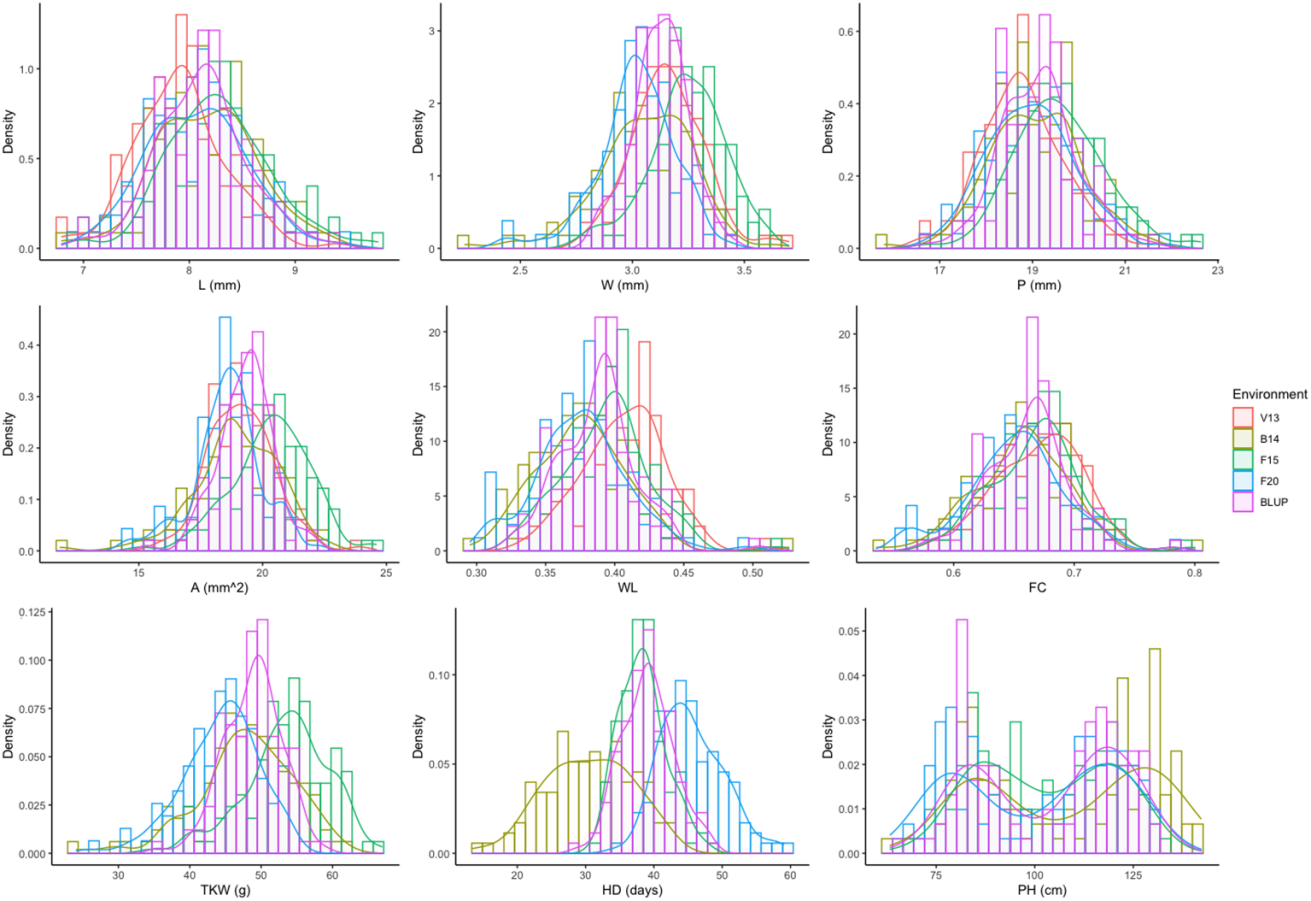
Frequency distribution for the nine phenotypic traits analyzed for each environment (V13, B14, F15, and F20) and BLUPs (overall data). Traits are denoted as L, length; W, width; P, perimeter; A, area; WL, width-to-length ratio; FC, form coefficient; TKW, thousand kernel weight; HD, heading days; and PH, plant height. For more details on the trait description, please refer to **Table 1** and the Materials and Methods section.

As depicted in **Figure 2**, the frequency distribution of phenotypic values for each trait in each environment and across environments suggested the contribution of several loci controlling the phenotypic variation for each trait (quantitative nature), including HD. The unique exception is PH, whose bimodal distribution indicated one major gene. In addition, high transgressive segregation was observed for all traits in both directions, including TKW, which implies the presence of superior alleles for the kernel-related traits in both parents.

The analysis of variance (one-way ANOVA) detected highly significant differences among RILs for all traits in each environment (*p*< 0.0001, **Appendix C**), which indicates that genetic factors explain a large fraction of the observed phenotypic variability. However, for F15, the replication factor was also significant and higher than the genotype factor, which could imply experimental error in experimental field; for this reason, the replication 1 was removed for the rest of the analysis. Variance components computed by BLUPs on the overall dataset across environments revealed the effects of RILs, environments, and GEI, as shown in **Table 3**. Variance of the environment (ENV) component ranges from 3.6% for PH to 68.5% for HD. Variance component related to genotype (GEN) ranged from 19.1% for DH to 78.6% for PH, with kernel L having the highest value among the kernel related traits and TKW having the lowest value. Variance of GEN component was greater than the GEI component (ranging from 7.2% to 26%) for all the traits considered in this study, suggesting that the genetic factors contributed largely to the phenotypic variability, as also shown by moderate to high heritability computed for all traits (**Table 3**).

**Table 3:** Percentage of variance components for random effects from the BLUP models for the 9 traits analyzed in the MG5323 x Latino RIL mapping population.

Source of variation	L	W	P	A	WL	FC	TKW	HD	PH
**GEN**	73.2	37.9	67.7	45.9	62.2	68.8	32.4	19.1	78.6
**ENV**	8.9	21.3	9.8	16.4	17.8	5.6	33.0	68.5	3.6
**GEN : ENV**	9.4	26.0	11.8	22.4	13.3	16.5	20.6	7.2	7.8
**REP(ENV)**	1.0	2.3	1.5	3.1	0.2	0.3	1.9	0.3	0.9
**Residuals**	7.5	12.6	9.2	12.2	6.5	8.7	12.0	4.9	9.1
**H^2^**	0.813	0.496	0.763	0.569	0.758	0.732	0.498	0.613	0.823

GEN, genotype effect; ENV, environment effect; GEN : ENV, genotype × interaction effect; REP(ENV), replicate effect within each environment; H^2^ is broad-sense heritability.

Traits are denoted as L, length; W, width; P, perimeter; A, area; WL, width-to-length ratio; FC, form coefficient; TKW, thousand kernel weight; HD, heading days; and PH, plant height. For more details on trait description, please refer to **Table 1** and the Materials and Methods section.

**Figure 3:**
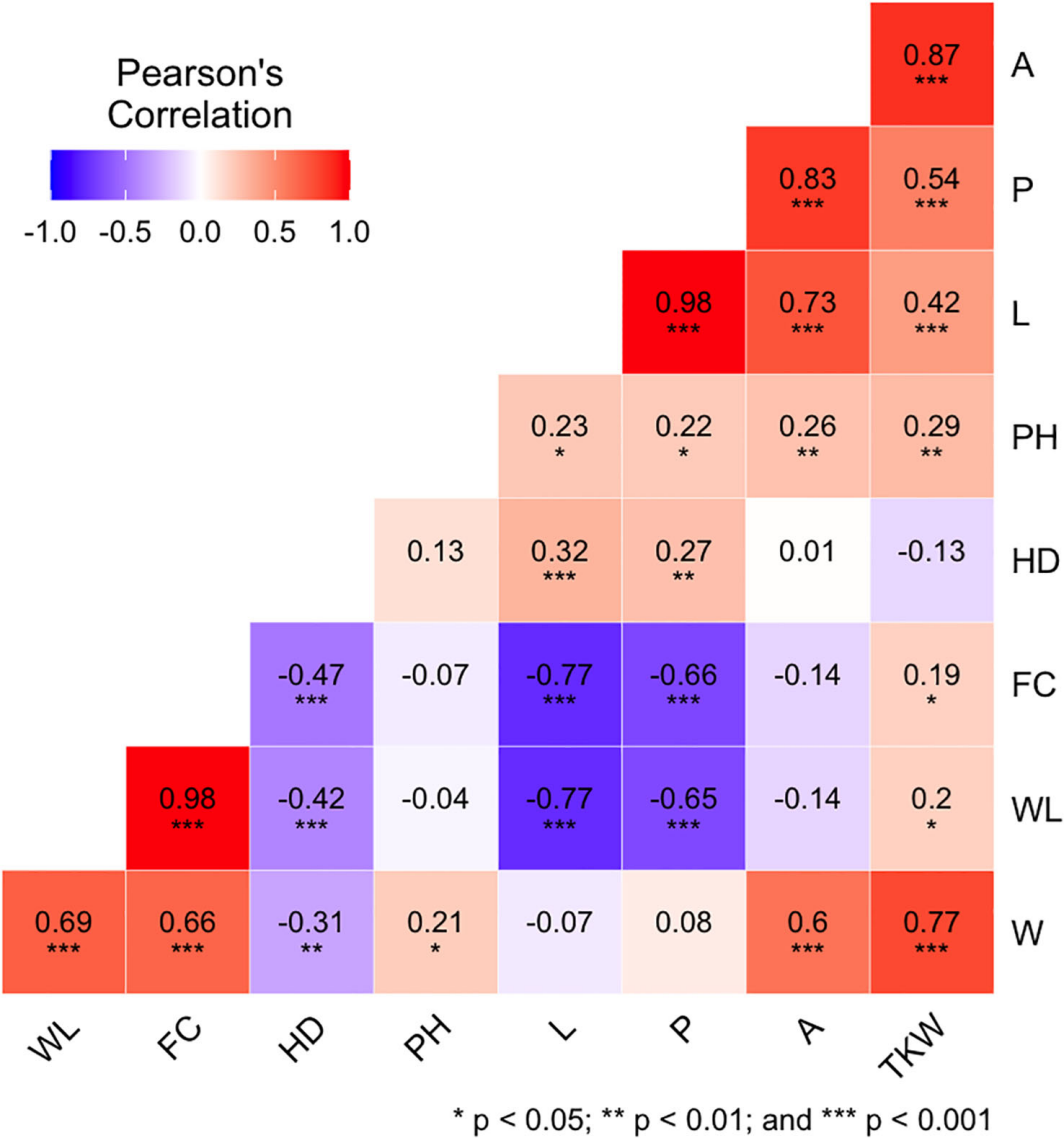
Pearson correlation coefficient (*r*) among the nine phenotypic traits analyzed using BLUPs (overall data). Traits are denoted as L, length; W, width; P, perimeter; A, area; WL, width-to-length ratio; FC, form coefficient; TKW, thousand kernel weight; HD, heading days; and PH, plant height. For more details on the trait description, please refer to **Table 1** and the Materials and Methods section.

Correlation analysis was performed for the phenotypic data of each environment (**Appendix D**) and for BLUPs (**Figure 3**) among the nine evaluated traits. These kernel-related traits can be distinguished in primary (L and W) and secondary (P, A, WL, and FC) being derived by combinations of the primary traits, leading to inherent correlation between them. In detail, for each environment and BLUPs, for kernel size traits, kernel L is the main feature related to its secondary features A (r ≈ 0.7) and P (r ≈ 1). Meanwhile, kernel W is the main trait for WL and FC attributes (r ≈ 0.7). Interestingly, TKW showed a significant and highly positive correlation to A (r ≈ 0.9) and W (r ≈ 0.8) and moderate positive correlation to L (r ≈ 0.4) and P (r ≈ 0.5) for all environments and BLUPs. The correlations between TKW and kernel shape traits were significant (*p*< 0.05) with r-values lower than the traits mentioned before. In addition, PH showed a moderate positive correlation with TKW and A (r ≈ 0.3) as well as with L, W, and P (r ≈ 0.2), whereas HD was positively correlated with L and P (r ≈ 0.3) and negatively correlated with W (r ≈ −0.3), WL (r ≈ −0.4), and FC (r ≈ −0.5).

### QTL analysis

QTL analysis was performed for all traits recorded in the four individual environments (V13, B14, F15, and F20) and on BLUPs, finding a total of 100 individual significant QTLs and five suggestive/putative QTLs (**Appendix E**). In the different environments, the number of QTLs identified was 15, 19, 24, and 18 in V13, B14, F15, and F20, respectively, whereas 24 QTLs were identified on BLUPs. The explained phenotypic variance ranged from 6.9% to 22%, with an average of 12.5% for kernel related size/weight traits and from 6.8% to for 42.7% for HD. The highest average explained variation was found for P (14.2%), whereas the lowest was calculated for FC (10.7%). QTLs for kernel traits were found on all chromosomes, with exception of chromosome group 1 and chromosomes 5B, 6A, and 7B. QTLs for HD were mostly distributed on chromosome groups 2 and 5, in addition to one QTL each on chromosomes 7B and 3A, whereas QTLs for PH were all on 4B.

Many QTLs were coincident or close together, suggesting that the same genomic region was the genetic determinant of the same trait in different environments and, thus, that stable QTLs were identified. Therefore, for each trait, QTLs whose peak markers were less than 10 cM faraway and/or have overlapping CIs were considered to correspond to the same QTL, provided that the additive effect was conferred by the same parent (**Table 4**). The 10-cM threshold was prudently chosen on the basis of the size of the largest QTL CI calculated for the 100 identified QTLs. An exception was made for QTLs for L, P, A, and TKW on chromosome 4B identified at 63 cM in F20. These QTLs were grouped with QTLs for the same traits identified at about 79–82 cM in B14, F15, and BLUPs, considering the greater consistency of these latter QTLs and imputing the shift due to the reduced number of RILs used in F20.

**Table 4:** Summary of the QTL detected in the MG5323 x Latino RIL mapping population for the nine traits analyzed for each environment and across environments (BLUP).

Trait	QTL name	Chr.	Peak Pos. Interval (cM)	Environments																				Across environments (BLUP)					stable QTL
				V13					B14					F15					F20										
				LOD	R2 (%)	Add	CIs cM	CIe cM	LOD	R2 (%)	Add	CIs cM	CIe cM	LOD	R2 (%)	Add	CIs cM	CIe cM	LOD	R2 (%)	Add	CIs cM	CIe cM	LOD	R2 (%)	Add	CIs cM	CIe cM	
**L**	QL-2A	2A	78.2-83.1	3.8	12.8	0.2	72.4	84.0	*2.8*	*8.1*	*0.1*	74.0	92.2	–	–	–	–	–	–	–	–	–	–	–	–	–	–	–	
	QL-2B	2B	121.1	–	–	–	–	–	–	–	–	–	–	5.5	15.4	0.2	116.3	125.9	–	–	–	–	–	–	–	–	–	–	
	QL-4B	4B	78.8-79.5*	–	–	–	–	–	3.5	10.3	0.2	72.3	86.7	4.9	13.3	0.2	73.2	84.4	5.8	18.6	0.2	59.42*	67.38*	4.1	13.5	0.2	74,0	85,0	x
	QL-6B	6B	49.6	–	–	–	–	–	–	–	–	–	–	4.0	10.6	-0.2	42.6	56.6	–	–	–	–	–	–	–	–	–	–	
	QL-7A	7A	77.4-90.6	4.2	14.2	0.2	76.0	86.4	3.7	10.8	0.2	78.8	92.4	3.6	9.7	0.2	83.0	98.2	6.7	22.0	0.2	74.0	80.8	3.7	12.2	0.1	79,5	91,7	x
	**Model**			7.0	**25.5**				10.3	**35.1**				12.6	**41.1**				10.3	**36.9**				7.6	**27.4**				
**W**	QW-2B	2B	31.2	–	–	–	–	–	–	–	–	–	–	–	–	–	–	–	3.1	10.8	-0.1	24.3	38.1	–	–	–	–	–	
	QW-3A	3A	77.4	–	–	–	–	–	–	–	–	–	–	–	–	–	–	–	3.5	12.4	-0.1	71.4	83.4	–	–	–	–	–	
	QW-4B	4B	21	4.0	13.1	0.1	15.3	26.7	–	–	–	–	–	–	–	–	–		–	–	–	–	–	–	–	–	–	–	
	QW-5A	5A	164.8	–	–	–	–	–	–	–	–	–	–	3.7	11.9	0.1	158.6	171.0	–	–	–	–	–	–	–	–	–	–	
	QW-6B	6B	66.5-67.1	–	–	–	–	–	3.8	14.6	-0.1	61.4	71.6	*3.0*	*9.4*	*-0.1*	59.2	75.0	–	–	–	–	–	–	–	–	–	–	x
	QW-7A	7A	77.4	5.0	16.5	-0.1	72.9	81.9	–	–	–	–	–	3.8	12.3	-0.1	71.4	83.4	–	–	–	–	–	3.8	14.5	0.0	72,3	82,5	x
	**Model**			8.1	**28.7**			** **	3.8	14.6				8.1	**28.7**				6.9	**26.4**				3.8	14.5				
**P**	QP-2A	2A	111.2	3.1	9.7	0.3	103.6	118.8	–	–	–	–	–	–	–	–	–	–	–	–	–	–	–	–	–	–	–	–	
	QP-2B	2B	43.2	3.1	9.7	0.3	35.6	50.8	–	–	–	–	–	–	–	–	–	–	–	–	–	–	–	–	–	–	–	–	
	QP-4A	4A	105.2	–	–	–	–	–	3.9	12.5	0.3	99.3	111.1	–	–	–	–	–	–	–	–	–	–	–	–	–	–	–	
	QP-4B	4B	79.5*	–	–	–	–	–	5.0	16.6	0.4	75.0	84.0	4.0	15.5	0.4	74.7	84.3	6.6	22.1	0.4	60.04*	66.76*	4.6	15.6	0.3	74,7	84,3	x
	QP-7A.1	7A	78.1-85.6	–	–	–	–	–	–	–	–	–	–	–	–	–	–	–	5.7	18.7	0.4	74.1	82.1	*2.9*	*9.4*	*0.3*	77,8	93,4	x
	QP-7A.2	7A	103.4	3.7	11.9	0.3	97.2	109.6	–		–	–	–	–	–	–	–	–	–	–	–	–	–	–	–	–	–	–	
	**Model**			8.3	**29.3**			** **	8.4	**29.5**				4.0	15.5				9.9	**35.8**				7.4	**26.6**				
**A**	QA-3B	3B	114.3	–	–	–	–	–	–	–	–	–	–	3.5	10.3	-0.5	107.0	121.6	–	–	–	–	–	3.4	9.9	-0.4	106,8	121,8	x
	QA-4A	4A	22.7	–	–	–	–	–	3.4	9.9	0.6	15.2	30.2	–	–	–	–	–	–	–	–	–	–	–	–	–	–	–	
	QA-4B	4B	79.5-81.5*	–	–	–	–	–	3.1	9.2	0.5	71.4	87.6	3.9	11.6	0.5	75.1	87.9	4.2	17.1	0.6	59.07*	67.73*	3.9	11.8	0.4	75,2	87,8	x
	QA-6B	6B	66.5	–	–	–	–	–	6.6	20.7	-0.8	62.9	70.1	4.1	12.3	-0.6	60.5	72.5	–	–	–	–	–	4.1	12.4	-0.4	60,5	72,5	x
	**Model**				* *			** **	10.2	**34.6**				10.1	**34.5**				4.2	17.1				10.1	**34.4**				
**WL**	QWL-2A	2A	43.4	–	–	–	–	–	–	–	–	–	–	–	–	–	–	–	3.5	8.5	0.0	34.7	52.1	–	–	–	–	–	
	QWL-2B	2B	114.9-122.4	3.8	9.7	0.0	114.8	130.0	3.4	11.0	0.0	108.2	121.6	3.6	11.4	0.0	115.9	128.9	3.1	7.6	0.0	111.3	130.9	4.2	10.8	0.0	115,5	129,3	x
	QWL-3A	3A	77.4	3.6	9.1	0.0	69.3	85.5	–	–	–	–	–	–	–	–	–	–	3.6	8.7	0.0	68.9	85.9	4.3	11.0	0.0	70,7	84,1	x
	QWL-7A	7A	77.4-86.9	7.4	20.3	0.0	75.7	82.9	4.2	14.0	0.0	72.8	83.4	4.9	15.8	0.0	72.7	82.1	4.9	12.2	0.0	80.1	92.3	6.0	15.9	0.0	82,2	91,6	x
	**Model**			13.8	**43.8**			** **	7.9	**28.1**				8.5	**30.0**				15.4	**49.8**				14.0	**44.4**				
**FC**	QFC-2A	2A	43.4	–	–	–	–	–	–	–	–	–	–	–	–	–	–	–	4.0	10.1	0.0	36.0	50.8	–	–	–	–	–	
	QFC-2B.1	2B	43.4	3.5	7.6	0.0	33.5	52.9	–	–	–	–	–	–	–	–	–	–	–	–	–	–	–	3.5	7.6	0.0	33,4	53,0	x
	QFC-2B.2	2B	114.9-122.4	3.2	6.9	0.0	104.2	125.6	3.8	12.2	0.0	108.8	121.0	3.3	10.3	0.0	115.2	129.6	*2.9*	*7.0*	*0.0*	113.7	128.5	4.5	10.0	0.0	107,5	122,3	x
	QFC-3A	3A	77.4	5.8	13.3	0.0	71.8	83.0	–	–	–	–	–	–	–	–	–	–	3.4	8.4	0.0	66.8	88.0	5.4	12.2	0.0	71,3	83,5	x
	QFC-7A	7A	77.4-86.2	5.7	12.9	0.0	73.5	85.1	4.4	14.2	0.0	72.9	83.3	5.4	17.6	0.0	73.2	81.6	4.4	11.1	0.0	77.4	95.0	4.5	9.9	0.0	70,6	85,6	x
	**Model**			17.6	**52.1**		** **	** **	8.5	**29.8**				8.8	**30.7**				15.2	**49.3**				17.4	**51.8**				
**TKW**	QTKW-3B.1	3B	114.3	Not available					–	–	–	–	–	4.0	12.8	-2.2	108.5	120.1	–	–	–	–	–	–	–	–	–	–	
	QTKW-3B.2	3B	154.9						–	–	–	–	–	–	–	–	–	–	–	–	–	–	–	4.2	13.3	-1.5	149.3	160.5	
	QTKW-4B.1	4B	21						–	–	–	–	–	3.3	10.4	2.0	13.8	28.2	–	–	–	–	–	–	–	–	–	–	
	QTKW-4B.2	4B	82.1*						–	–	–	–	–	–	–	–	–	–	3.0	12.6	1.9	57.54*	69.26*	3.1	9.6	1.3	74.3	89.9	x
	QTKW-6B	6B	66.5-67.1						4.0	15.4	-2.6	62.3	71.9	3.5	11.0	-2.0	59.8	73.2	–	–	–	–	–	4.3	13.6	-1.5	61.7	72.5	x
	**Model**								4.0	15.4				8.5	**29.9**				3.0	12.7				8.7	**30.4**				
**HD**	QHD-2A	2A	34.9-35.6						17.3	42.8	4.1	33.2	36.6	5.2	9.6	1.1	27.9	43.3	–	–	–	–	–	13.2	26.1	1.9	32.1	37.7	x
	QHD-2B	2B	46.4						*2.8*	*5.0*	*1.4*	31.5	61.3	5.7	10.6	1.2	39.4	53.4	9.0	27.8	2.5	43.7	49.1	6.6	11.3	1.3	39.8	53.0	x
	QHD-3A	3A	5.6						–	–	–	–	–	3.9	6.9	1.0	0.0	16.3	–	–	–	–	–	–	–	–	–	–	
	QHD-5A	5A	124.4						5.0	9.3	1.8	116.4	132.4	–	–	–	–	–	–	–	–	–	–	4.9	8.1	1.1	115.2	133.6	x
	QHD-5B	5B	85.2						–	–	–	–	–	5.2	9.6	1.2	77.5	92.9	–	–	–	–	–	4.2	6.8	1.1	74.3	96.1	x
	QHD-7B	7B	39.9-48.1						3.9	7.0	1.5	37.6	58.6	6.9	13.2	1.2	36.5	47.7	4.1	11.4	1.6	33.4	46.4	7.0	12.1	1.3	42.0	54.2	x
	**Model**			21.8	**59.8**				22.3	**60.6**				11.9	**39.2**				24.7	**64.5**									
**PH**	QPH-4B	4B	25.6	22.1	71.1	18.6	24.6	26.6	18.3	64.2	13.1	24.4	26.8	21.4	70.0	16.4	24.5	26.7	Not available					24.2	63.7	13.8	24.4	26.8	x
	**Model**				Not available					Not available					Not available										Not available				

(-), Not significant; CIs and CIe: confidence intervals for start and end respectively; LOD, logarithm of odds; R^2^, percentage of the phenotypic variance explained; Add, additive effect of a QTL, where the absence of sign indicates alleles from parent MG5323, which are increasing the trait scores, whereas the negative sign (−) indicates alleles from parent Latino. Note: (*) For the QTLs on 4B for all traits (A, L, P, and TKW), the peak position in the F20 environment was located at 63 cM, whereas, in the rest of environments and across data, it was located at about 79–82 cM.

The best models with explained phenotypic variation over 25% are reported in bold. Suggestive/putative QTLs (LOD< 3.0) are reported in italics. Traits are denoted as L, length; W, width; P, perimeter; A, area; WL, width-to-length ratio; FC, form coefficient; TKW, thousand kernel weight; HD, heading days; and PH, plant height. Environment acronyms are as follows: V13, Valenzano 2012–2013; B14, Bologna 2013–2014; F15, Fiorenzuola d’Arda 2014–2015; and F20, Fiorenzuola d’Arda 2019–2020. For more details on trait and environment description, please refer to **Table 1** and the Materials and Methods section.

After grouping, a total of 42 different QTLs were defined (**Table 4**), and, among them, 24 were identified in at least one environment and by BLUPs and therefore considered environmentally stable. The 42 QTLs were distributed on 11 of the 14 chromosomes of the MG5323 x Latino linkage map. More in detail, for the kernel size traits (L, W, P, and A), a total of 21 QTLs were identified on chromosomes 2A, 2B, 3A, 3B, 4A, 4B, 5A, 6B, and 7A. Overall, nine loci were detected for kernel shape traits (WL and FC), located in chromosomes 2A, 2B, 3A, and 7A. For TKW, five QTLs were identified: two located on chromosome 3B, two on chromosome 4B, and one on 6B. For HD, six QTLs were found, mapped on chromosomes 2A, 2B, 3A, 5A, 5B, and 7B. For PH, only a major QTL on 4B was found. About the effect, for 23 of the 42 QTLs, the allele with a positive additive effect was contributed by the parent MG5323. In detail, four QTLs associated to L, two to W, six to P, two to A, two to TKW, six to HD, and one to PH. Meanwhile, the parental Latino carried all the alleles for increasing kernel shape traits (WL and FC) and thus for conferring more roundness to the kernels. In all the QTLs detected for HD, the alleles with a positive effect were contributed by the parent MG5323, which is indeed the late parent. Major/moderate QTLs (with R^2^ above 15%) were found, including three for L (QL-2B, QL-4B, and QL-7A), one for W (QW-7A), two for P (QP-4B and QP-7A.1), two for A (QA-4B and QA-6B), one for WL (QWL-7A), one for FC (QFC-7A), one for TKW (QTKW-6B), and two for HD (QHD-2A and QHD-2B). No significant epistatic interactions were identified in this study.

The 24 stable QTLs were on the chromosomes 2A, 2B, 3A, 4B, 6B, 7A, and 7B, and they were the following: QL-4B, QL-7A, QW-6B, QW-7A, QP-4B, QP-7A.1, QA-3B, QA-4B, QA-6B, QWL-2B, QWL-3A, QWL-7A, QFC-2B.1, QFC-2B.2, QFC-3A, QFC-7A, QTKW-4B.2, QTKW-6B, QHD-2A, QHD-2B, QHD-5A, QHD-5B, QHD-7B, and QPH-4B (**Table 4**). Interestingly, the most stable regions, identified in all environments and across them, were located on chromosomes 7A and 2B (QL-7A, QWL-2B, QWL-7A, and QFC-7A), in addition to QHD-2B, QHD-7B, and QPH-4B, which were identified in three environments and by BLUPs. The trait with the poorest stability was kernel W, where five of the six QTLs identified were only detected in one or two environments.

### QTL clusters

Because of the geometrical or biological nature of the relationships between the traits under study, co-location of QTLs for different traits was expected, as also suggested by Pearson’s correlation analysis. This implied the pleiotropic effect of a single gene or a set of linked genes on multiple related traits. Thus, 83 QTLs of the initial 100 were grouped into nine different QTL clusters, defined as regions where QTLs for different traits co-located, with their CIs being fully or partially overlapping (**Table 5**). Furthermore, the genetic position on the tetraploid wheat consensus map (Maccaferri et al., 2015) for each QTL of the nine cluster was obtained by projecting the molecular markers of the CIs, corroborating the co-location of these loci. The nine clusters identified were located on chromosomes 2A (cluster 1), 2B (clusters 2 and 3), 3A (cluster 4), 3B (cluster 5), 4B (clusters 6 and 7), 6B (cluster 8), and 7A (cluster 9).

**Table 5:** Description of QTL clusters. For each cluster, the cluster ID, traits and no. of individual QTLs involved, environments where QTL were identified, chromosomes, and donor parents are reported.

QTL Cluster	Traits involved	no: individual QTL in cluster	Environment where mentioned QTLs were identified	Chr.	Donor	Position on Latino x MG5323 map (cM)			Position on durum consensus map (cM)		Position on Svevo.v1 (Mbp)		Candidate Genes		
													Candidate Gene ID or N° of underlined genes	Start position (Mbp)	Function (Known genes)
						Peak	Left CI	Right CI	Left CI	Right CI	Start	End			
**1**	HD	5	B14, F15, and BLUP	2A	MG	43.4	36.0	50.8	43.5	54.6	34.1	48.3	Ppd-A1	36.6	Photoperiodism
	WL, **FC**		F20 for WL and FC		Latino										
**2**	HD, P	8	B14, F15, F20, and BLUP for HD	2B	MG	31.2	24.4	38.1	16.1	34.0	20.1	33.4	Ppd-B1	56.3*	Photoperiodism
			V13 for P										TRITD2Bv1G019940	43.1*	Cell proliferation
	**W**, FC		F20 for W		Latino										
			V13 and BLUP for FC												
**3**	L,	11	F15 for L	2B	MG	114.9	107.5	122.3	112.3	128.0	537.6	629.3	564 Genes	–	–
	WL, **FC**		ALL for WL and FC		Latino										
**4**	W, WL, **FC**	7	F20 for W	3A	Latino	77.4	71.3	83.5	65.9	77.8	439.3	534.9	576 Genes	462.0	Regulation of cell division and elongation (*D61*)
			V13, F20, and BLUP for WL and FC												
**5**	A, **TKW**	3	F15 and BLUP for A	3B	Latino	114.3	108.5	120.1	129.6	138.9	691.3	741.6	TRITD3Bv1G229090	695.9	Response to auxin
													TRITD3Bv1G229910	698.3	
			F15 for TKW										TRITD3Bv1G235190	717.9	
													TRITD3Bv1G231370	702.8	Regulation of cell division
													TRITD3Bv1G239650	729.7	Ubiquitination and auxin regulation
**6**	PH, **W,** TKW	6	ALL for PH	4B	MG	21.0	15.3	26.7	15.5	34.0	13.4	31.8	Rht-B1	29.3	Gibberellin insensitive dwarfing gene
			V13 for W												
			F15 for TKW												
**7**	L, **P**, A, TKW	14	B14, F15, F20, and BLUP for L, P, and A	4B	MG	79.5	75.0	84.0	77.0	84.9	594.7	619.2	TRITD4Bv1G175480	595.1	Auxin regulation
													TRITD4Bv1G179270	605.8	
													TRITD4Bv1G177190	600.5	Regulation of cell proliferation
			F20 and BLUP for TKW										TRITD4Bv1G171270	582*	Auxin transport and seed growth regulation (*BIG GRAIN PROTEIN 1*)
**8**	L, W, **A**, TKW	9	F15 for L	6B	Latino	66.5	62.9	70.1	73.4	80.9	263.2	467.3	582 Genes	300.8	Regulation of cell growth (*GW2*)
			B14 and F15 for W											373.4	Regulation of cell division (*FUWA*)
			B14, F15, and BLUP for A and TKW												
**9**	L, P,	20	ALL for L	7A	MG	79.3	75.7	82.9	92.9	107.9	113.9	167.0	TRITD7Av1G052720	117.2	Regulation of cell growth and differentiation
			F20 and BLUP for P										TRITD7Av1G055870	125.6	
	W, **WL**, FC		V13, F15, and BLUP for W		Latino								TRITD7Av1G050690	111.5*	Sucrose metabolism (*TaSus1*)
			ALL for WL and FC										TRITD7Av1G071860	168.5*	Heat acclimatation (*TaGASR7-A1*)

(-), Not available. (*), Near positions but not within the found physical intervals. For physical intervals higher than 60 Mbp, only the quantity of genes within and known genes are shown. 1 Mbp (Megabase pair) = 1,000,000 bp (base pairs). The start position of candidate genes refers to its position on the reference genome.

In addition, genetic position on the Latino x MG5323 linkage map and on the durum wheat consensus map (Maccaferri et al., 2015) and physical positions on the durum wheat reference genome Svevo.v1 are included. Candidate genes of QTL clusters detected in the MG5323 x Latino RIL mapping population for kernel-related traits are indicated together with their name or Id, position on Svevo.v1 and biological function. Best QTLs selected by the highest LOD and R^2^ within the cluster are shown in bold; for these QTLs, the positions were retrieved and reported. Traits are denoted as L, length; W, width; P, perimeter; A, area; WL, width-to-length ratio; FC, form coefficient; TKW, thousand kernel weight; HD, heading days; and PH, plant height. Environment acronyms are as follows: V13, Valenzano 2012–2013; B14, Bologna 2013–2014; F15, Fiorenzuola d’Arda 2014–2015; and F20, Fiorenzuola d’Arda 2019–2020. For more details on trait and environment description, please refer to **Table 1** and the Materials and Methods section.

Clusters 1 and 2, constituted by five and eight QTLs, respectively, were the only ones found related to HD and shape/size kernel traits (WL, FC, P, and W). Clusters 3, 4, and 9 included 11, 7, and 20 QTLs, respectively, and highlighted the expected geometrical relationship between main traits and their derivative ones. Most of these clusters indicated independence of L and W traits, except for cluster 9 where QTLs for both traits were detected. Meanwhile, clusters 5, 6, 7, and 8, composed of 3 to 14 different QTLs, were associated with kernel size/shape and TKW. Two major and consistent clusters were found on chromosome 4B, associating PH, W, and TKW in cluster 6 as well as L, P, A, and TKW in cluster 7. In both clusters, the positive alleles of all the QTLs were derived from the emmer parent (MG5323). This was coherent with the high transgressive segregation of the TKW observed in the RIL population, despite the lower TKW value of MG5323. Notably, in cluster 7, the QTLs explained from 9.6% to 12.7% of the kernel weight variance and up to 22% of the kernel size traits (L, P, and A) variation.

**Figure 4:**
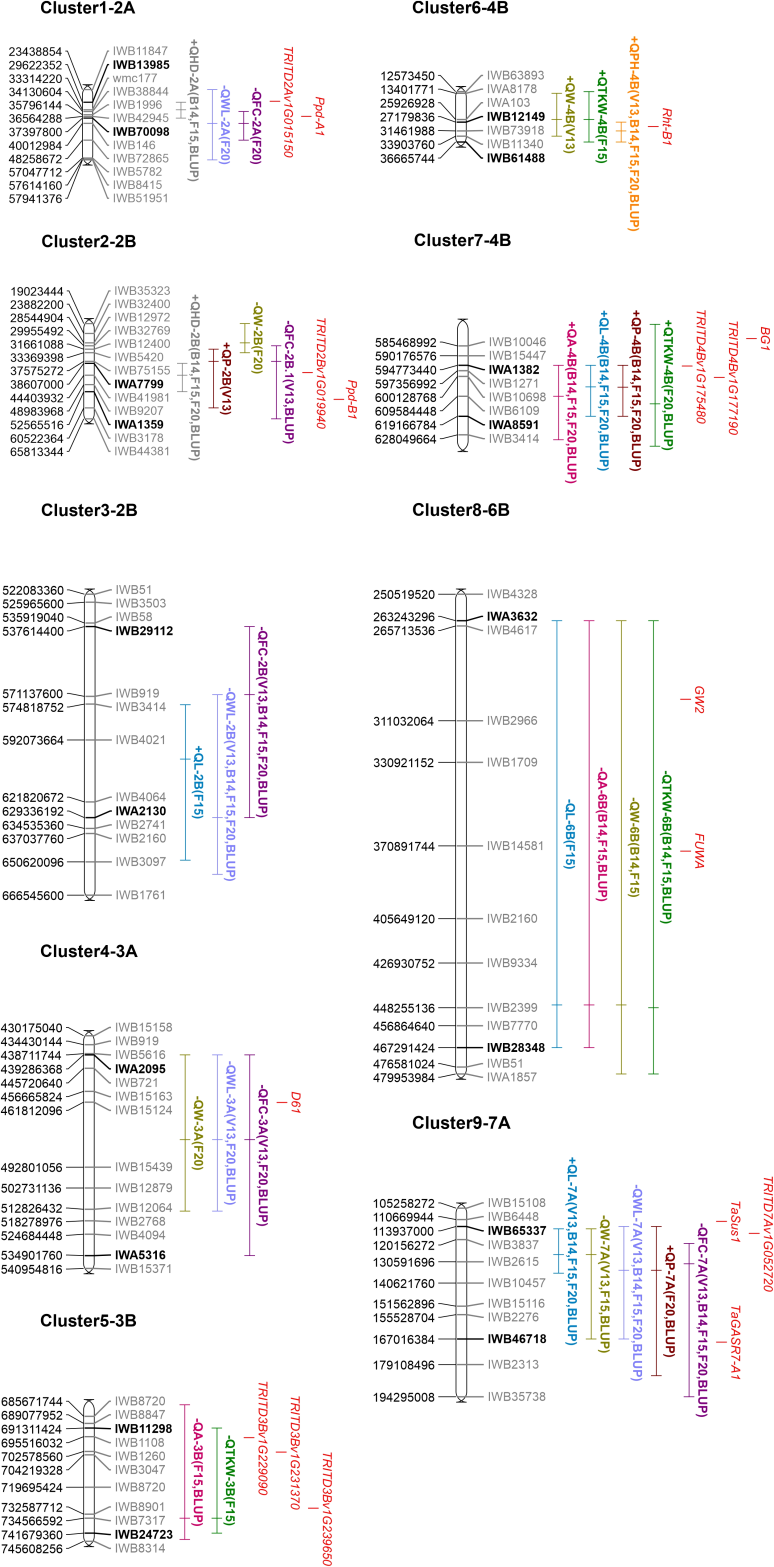
Schematic representation of clusters of QTL anchored on the durum wheat reference genome (created using MapChart version 2.3). Part of the chromosomes are represented by including some markers surrounding the QTL clusters; SNP marker IDs are on the right, whereas their positions on the durum wheat reference genome are reported in bp on the left. The name of flanking markers of the cluster intervals (major QTL of each cluster) is in bold. QTL names are according to **Table 4**. Traits are denoted as L, length; W, width; P, perimeter; A, area; WL, width-to-length ratio; FC, form coefficient; TKW, thousand kernel weight; HD, heading days; and PH, plant height. For more details on the trait description, please refer to **Table 1** and the Materials and Methods section. The + or − signs preceding the QTL name indicate the positive or negative additive effect of the allele carried by the parental line MG5323. Environments where reported QTLs were identified are also indicated in parentheses. Known and candidate genes hypothesized are shown in red. Complete information of this figure is on **Tables 5** and **6**.

The availability of the durum wheat reference genome (Svevo.v1; Maccaferri et al., 2019) allowed to define the physical interval of the clusters identified. To this aim, the best QTLs (QTL with the highest LOD and R^2^) related to size/shape traits within each cluster were projected on the Svevo genome. In this way, the largest physical regions were detected on chromosomes 2B (cluster 3), 3A (cluster 4), and 6B (cluster 8), which spanned for more than 90 Mbp (119 Mbp, 95 Mbp, and 216 Mbp, respectively). Clusters 1 and 2 spanned approximately 10 Mbp, clusters 5 and 9 for about 50 Mbp, whereas the two clusters, 6 and 7, identified on chromosome 4B spanned for 18 Mbp and 25 Mbp, respectively (**Figure 4**, **Table 5**).

### Identification of possible candidate genes for the QTL clusters

Candidate genes were hypothesized by inspecting the functional annotations [Gene Ontology (GO) terms] of the high-confidence Svevo genes retrieved within and/or near the physical intervals of the major QTL for each cluster (**Table 5**). Most attention was addressed to genes with GO terms likely associated with functions hypothetically related to kernel development and grain yield based on previous knowledge (i.e., hormone pathways and sugar metabolism). Then, an updated list of known genes controlling kernel related traits and kernel weight previously described in rice and/or wheat was also considered (**Appendix B**). Clusters 3, 4, and 8 were characterized by a large physical interval with a high number of annotated genes (around 500 for each cluster), making impossible the manual inspection of each gene under the CI of each QTL; therefore, only the comparison with known genes from **Appendix B** was carried out. The overall results are reported in **Appendix E** and represented in **Figure 4**.

About 300 Gb of 150-bp Illumina paired-end reads were obtained and aligned against the durum genome Svevo.v1 (Maccaferri et al., 2019), obtaining an average sequencing depth of 24.7 and a mean genome coverage of 98.7% (**Appendix F**). Overall, 11,414,704 genome-wide DNA variants were detected between MG5323 and the reference durum wheat genome, which were inspected to support the role of selected candidate genes. Assumption to this analysis is an extensive genetic similarity between the genome of the *cvs*. Latino and Svevo. Indeed, Latino and Svevo showed 85% similarity when genotyped with the Illumina iSelect 90K wheat array, whereas the similarity was 46% between both Latino and Svevo in respect to MG5323 (Mazzucotelli et al., 2020). Therefore, we used the Svevo.v1 genome assembly as surrogate of the cv. Latino genome, and we supposed that polymorphisms (SNPs or small INDELs) in corresponding genes between Svevo and MG5323 were likely conserved between Latino and MG5223. Notably, as a *de novo* assembly of MG5323 genome was not achievable, we cannot exclude that larger structural variations at candidate genes underlied the target traits. The analysis focused on gene sequences and upstream regions (2,000 bp) of the 15 candidate genes, which included both the novel proposed candidates (*TRITD2Bv1G019940*, *TRITD3Bv1G229090*, *TRITD3Bv1G229910*, *TRITD3Bv1G235190*, *TRITD3Bv1G239650*, *TRITD4Bv1G175480*, *TRITD4Bv1G179270*, *TRITD7Av1G052720*, *TRITD7Av1G055870*) and some known cloned genes (*D61*, *TRITD3Av1G163790; BG1*, *TRITD4Bv1G171270; GW2*, *TRITD6Bv1G096950; FUWA*, *TRITD6Bv1G115800; TASUS1*, *TRITD7Av1G050690; TAGASR7*, and *TRITD7Av1G071860*) located in the CI of the identified QTLs (**Table 5**, **Figure 4**). A total of 67 SNPs between orthologous sequences of MG5323 and Svevo genomes were identified: 46 in the upstream regions and 21 in the gene sequences (**Appendix G**). No SNPs were identified for *TRITD2Bv1G019940* and *TRITD6Bv1G115800* (*FUWA*). The highest number of SNPs in the upstream region was identified in *TRITD3Bv1G235190* and *TRITD7Av1G055870* (9 and 11, respectively). Considering the gene sequences, only eight genes reported SNPs, and, among them, only three genes (*TRITD3Bv1G229090*, *TRITD3Bv1G229910*, and *TRITD3Bv1G235190*) had SNPs in the coding sequence (two, two, and seven SNPs, respectively). Seven of them were synonymous variants, whereas the remaining four (two in *TRITD3Bv1G229090*, one in *TRITD3Bv1G229910*, and one in *TRITD3Bv1G235190*) were classified as missense variants. Three of them changed the aminoacidic chemical characteristics (Leu/Gln, Glu/Gln, and Val/Ile) with a possible consequence on the protein folding and/or activity.

**Figure 5:**
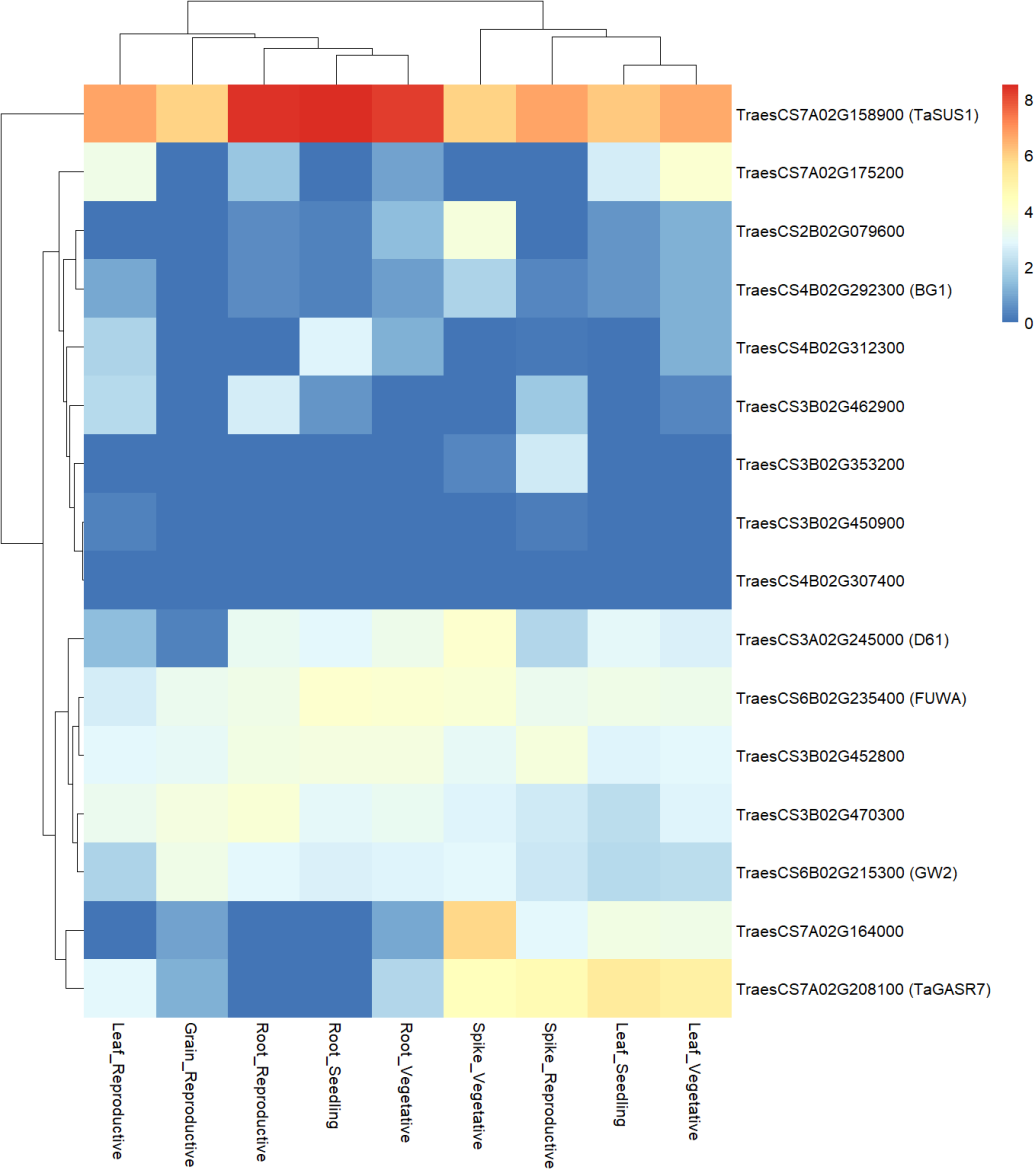
Heatmap of gene expression of bread wheat homologs of QTL candidate genes. Expression level of each gene in different organs and at different developmental stage is reported, as obtained from ExpVIP database. Gene expression levels are expressed as log2 of transcript abundances, normalized as for transcript per million (tpm). Expression level is shown according to the color scale reported, from blue for no expression, to red for highest expression.

Upon identification of orthologs of candidate genes in bread wheat, gene expression atlas available for bread wheat through ExpVIP was inspected to gain some functional evidence to support the candidates. Bread wheat homologous genes were identified for all candidates (**Appendix G**) but one (*TRITD4Bv1G175480*). The expression profile of these genes was *in silico* analyzed for different plant organs and developmental stages, considering both relevant (spikes, grains, and reproductive stage) and not relevant (leaves, roots, and vegetative stage) plant tissues (**Figure 5**). The most expressed gene was *TaSUS1*/*TraesCS7A02G158900*, and, then, other genes can be classified in three different groups according to the general expression profile. One group contained genes with general medium expression level (*D61/TraesCS3A02G245000*, *FUWA/TraesCS6B02G235400*, *GW2/TraesCS6B02G215300*, *TraesCS3B02G470300*, and *TraesCS3B02G452800*), a second group included genes with general low expression level (*BG1/TraesCS4B02G292300*, *TraesCS2B02G079600*, *TraesCS3B02G450900*, *TraesCS3B02G353200*, *TraesCS3B02G462900*, *TraesCS4B02G312300*, *TraesCS7A02G175200*, and *TraesCS4B02G307400*), lastly there were a couple of genes (*TaGASR7/*TraesCS7A02G208100 and *TraesCS7A02G164000*) with higher expression into specific organs (leaf and spikes). Of particular interest is the expression profile of some unknown candidate genes that showed a specific induction in spikes at vegetative (*TraesCS2B02G079600* and *TraesCS7A02G164000*) or reproductive plant stage (*TraesCS3B02G353200*).

## Discussion

Unraveling the genetic bases determining yield components, such as TKW, is an ongoing and essential task to drive grain yield improvement. In this way, attention should be paid to kernel size and shape factors, which are important parameters for grain weight and have been manipulated because of domestication, selection, and improvement for grain yield. The molecular mechanisms behind these traits have been mainly studied in bread wheat, whereas, in durum wheat, there is still a huge terrain to cover (Desiderio et al., 2019; Mangini et al., 2021; Haugrud et al., 2023). Moreover, wheat ancestors as cultivated emmer (*T. dicoccum*) should be considered as promising genetic resources to be employed for studying effects of genetic improvement and restoring durum wheat diversity (Rahman et al., 2020; Mohammadi et al., 2021). Under this context, the present study was conceived to dissect the genetic network behind kernel size and shape traits, kernel weight, PH, and HD, by performing QTL mapping on a RIL population derived from a *T. dicoccum* accession.

### Detection of environmentally stable QTLs, trait relationships, and favorable alleles from *T. dicoccum* MG5323

In this study, the ANOVA across four environments (location–year) showed that the genotypic effect was higher than the GEI effect for all traits. Thus, we were able to detect environmentally stable QTLs (24) for most of the kernel morphological traits, which implied their reliability in the determination of the considered traits. Kernel W was the most unstable trait. This could imply that kernel W might be controlled by minor effect genes under a relatively higher environmental effect, as exposed in two previous studies in durum wheat, where low heritability was also detected for this trait (Desiderio et al., 2019; Sun et al., 2020).

Co-locating loci defined nine QTL clusters. Some of them were expected (clusters 3, 4, and 9) as a consequence of inherent geometrical relationships between main kernel traits and their mathematically derivative ones, also suggested by the Pearson’s correlation coefficients. The relationship between kernel L and W is more intriguing. Indeed, the identification of associated regions that independently control these two kernel traits might allow the use of this genome-based information to obtain the target kernel ideotype. On the other hand, one QTL determining both traits may allow one to focus on only one genomic region to efficiently increase kernel A. In this study, as previously shown, W and L were found controlled by different clusters, and no significant correlation was identified in the correlation analysis, so the independence of both traits could be implied as in previous studies (Desiderio et al., 2019; Mangini et al., 2021). The only exception was represented by the cluster 9, on chromosome 7A, which included QTLs with positive allelic effects provided by both parents. These findings suggest that genes controlling the traits are closely linked and could allow their exploitation to parallelly increment them.

The highest significant positive relationship between a kernel size trait and TKW was detected from Pearson’s correlation analysis for kernel A (*r* ≈ 0.9) and further confirmed by coincident loci detected for both traits in clusters 5, 7, and 8. There is compelling and expected evidence for this relationship, suggesting that TKW improvement is due to the kernel A increase (Russo et al., 2014; Desiderio et al., 2019; Sun et al., 2020; Mangini et al., 2021). Moreover, these co-located loci confirmed the expectation that gene(s) responsible for variation of kernel size/shape might also affect kernel weight. Some recent examples about this assumption have been documented in both bread and durum wheat (Avni et al., 2018; Desiderio et al., 2019; Wang et al., 2019; Xin et al., 2020; Li et al., 2021; Mangini et al., 2021; Qu et al., 2021), and, in some cases, it has been also confirmed by QTL cloning in rice and wheat (Yamamuro et al., 2000; Chen et al., 2015; Zhang et al., 2017). The other significant positive relationship was obtained between kernel W and TKW (*r* ≈ 0.8), which was confirmed by clusters 6 and 8; however, the environmental dependency of the QTLs related to kernel W, as explained before, needs to be taken in account for further studies on these relationships.

Two clusters, 1 and 2, on chromosomes 2A and 2B, respectively, grouped QTLs for kernel shape/size traits and HD, highlighting well-known ectopic effects of plant phenology on yield components (Wilhelm et al., 2009). The study by Mangini et al. (2021), using a RIL population derived from a cross between two durum wheat lines, also reported about a cluster on chromosome 2A associated with HD and kernel traits; however, it included kernel A and kernel L, a relationship not found in this work, which might be due to the difference of genetic backgrounds.

The two clusters on 4B (6 and 7) are of major interest because they included TKW beside kernel size traits, with favorable alleles originated from MG5323. This result is consistent with several studies supporting *T. dicoccum* as donor of valuable alleles to increase seed size and weight (Thanh et al., 2013; Faris et al., 2014; Russo et al., 2014; Wang et al., 2019), whereas Guan et al. (2018) referred to wheat chromosome 4B as a “QTL-hotspot,” thus a shared genomic region with a pleiotropic effect or tightly linked loci affecting two or more traits. Noteworthy, about cluster 7, this study suggests that L is the main trait contributing to A, which implies that the increase of kernel A through L could be achievable using a *T. dicoccum* line for durum wheat breeding.

### Comparative analysis of QTL clusters

The comparison of physical positions of the clusters detected in this study with QTLs from previous studies (from both linkage and association mapping; **Appendix A**) was performed to assess the novelty of our results (**Table 6**) through a first projection on the consensus map as bridge and then on the reference genome. Most of the QTLs identified in this work fell within regions previously identified for kernel-related traits, despite different genetic backgrounds and experimental conditions. Nonetheless, this study allowed incrementing the number of traits associated to each of the co-locating QTL.

**Table 6:** Co-location of previously reported QTLs for kernel-related traits with QTLs reported in the present study.

Reference	Trait	Tetraploid wheat cross population or collection	Chr.	Left marker on Svevo.v1		Right marker on Svevo.v1	
				Marker ID	Physical position (Mbp)	Marker ID	Physical position (Mbp)
**This study: Cluster 1**	**HD. WL. FC**	**MG5323 (*T. dicoccum*) x Latino**	2A	**IWB38844**	**34.1**	**IWB72865**	**48.3**
Graziani et al. (2014)	TKW	Kofa x Svevo		IWA5087	32.5	IWB72463	42.6
**This study: Cluster 2**	**HD. P. W. FC**	**MG5323 (*T. dicoccum*) x Latino**	2B	**IWB65752**	**20.05**	**IWB2316**	**33.4**
Patil et al. (2013)	*TKW*	PDW233 x Bhalegaon 4		*IWA7916*	*53.4*	*IWB44381*	*64.0*
**This study: Cluster 3**	**L. WL. FC**	**MG5323 (*T. dicoccum*) x Latino**	2B	**IWB29112**	**537.6**	**IWA2130**	**629.3**
Desiderio et al. (2019)	L. WL	Iran_249 x Zardak		IWB39200	448.4	IWB69139	546.4
**This study: Cluster 4**	**W. WL. FC**	**MG5323 (*T. dicoccum*) x Latino**	3A	**IWA2095**	**439.3**	**IWA5316**	**534.9**
Avni et al. (2018)	TKW	Svevo x Zavitan (*T. dicoccoides*)		IWB16112	487.2	IWB20961	521.7
Blanco et al. (2012)	*TKW*	Ciccio x Svevo		*IWB66938*	*543.7*	*IWB44737*	*568.7*
Sun et al. (2020)	TKW	Worldwide collection		N/A^a^	419.1	N/A	521.1
Wang et al. (2019)	W	Worldwide collection		N/A^b^	447.5	N/A	466.7
Wang et al. (2019)	W	Worldwide collection		N/A^c^	448.0	N/A	467.2
**This study: Cluster 5**	**A. TKW**	**MG5323 (*T. dicoccum*) x Latino**	3B	**IWB11298**	**691.3**	**IWB24723**	**741.6**
Desiderio et al. (2019)	*TKW*	Iran_249 x Zardak		*IWB9399*	*781.1*	*IWB71782*	*817.5*
Faris et al. (2014)	TKW	Ben (PI596557) x PI 41025 (*T. dicoccum*)		IWA5510	741.1	IWA1094	778.4
Mangini et al. (2018)	TKW	Modern/old durum cvs. durum landraces and wild		wPt-7145	742.6	IWA1745	774.1
**This study: Cluster 6**	**PH. W. TKW**	**MG5323 (*T. dicoccum*) x Latino**	4B	**IWA2125**	**13.4**	**IWB71276**	**31.8**
Iannucci et al, (2017)	PH	Simeto x Molise Colli (*T. dicoccum*)		IWB72203	26.6	IWB72936	51.0
Russo et al. (2014)	W. A	Simeto x Molise Colli (*T. dicoccum*)		IWB73001	25.0	IWB72936	51.0
Russo et al. (2014)	TKW	Simeto x Molise Colli (*T. dicoccum*)		IWB7142	21.9	IWB72936	51.0
Patil et al. (2013)	TKW	PDW233 x Bhalegaon 4 (*T. durum* landrace)		IWB56078	30.6	IWB35104	48.3
Soriano et al. (2017)	TKW	Collection of mediterranean landraces		IWB58052	27.1	IWB69705	180.0
**This study: Cluster 7**	**A. L. P. TKW**	**MG5323 (*T. dicoccum*) x Latino**	4B	**IWA1382**	**594.7**	**IWA8591**	**619.2**
Blanco et al. (2012)	TKW	Ciccio x Svevo		IWA2398	555.1	IWB59718	582.3
Elouafi et al. (2004)	TKW	Omrabi5/*T. dicoccoides* 600545//Omrabi5 (BC1F8)		IWB34975	501.2	IWB8082	599.3
Graziani et al. (2014)	*TKW*	Kofa x Svevo		*IWB71667*	*629.0*	*IWB32544*	*654.0*
Mangini et al. (2021)	A. W	Liberdur x Anco Marzio		IWB38381	567.5	IWB17082	598.5
**This study: Cluster 8**)	**A. W. TKW**	**MG5323 (*T. dicoccum*) x Latino**	6B	**IWA3632**	**263.2**	**IWB28348**	**467.3**
Desiderio et al. (2019)	A	Iran_249 x Zardak		IWB5586	150.1	IWB73148	449.7
Tzarfati et al. (2014)	TKW	Langdon x G18-16 (*T. dicoccoides*)		IWB58306	443.0	IWB73374	562.8
Sun et al. (2020)	WL	Worldwide collection		N/A^d^	301.4	N/A	403.4
**This study: Cluster 9**	**L. W. WL. P. FC**	**MG5323 (*T. dicoccum*) x Latino**	7A	**IWB65337**	**113.9**	**IWB46718**	**167.0**
Desiderio et al. (2019)	*FC*	Iran_249 x Zardak		*IWB53096*	*673.0*	*IWB39743*	*673.0*
Patil et al. (2013)	TKW	PDW233 x Bhalegaon 4		IWB14901	106.1	IWB47160	123.3
Sun et al. (2020)	W	Worldwide collection		N/A^e^	106.6	N/A	208.6

For previously reported QTLs, the corresponding reference, trait, mapping population/germplasm collection, and flanking markers with position (in Mbp) on the reference genome Svevo.v1 are reported. Clusters found in this study are shown in bold. Nearby QTLs but not overlapped to the cluster’s positions are shown in italics. Traits are denoted as L, length; W, width; P, perimeter; A, area; WL, width-to-length ratio; FC, form coefficient; TKW, thousand kernel weight; HD, heading days; and PH, plant height. For more details on trait description, please refer to **Table 1** and the Materials and Methods section.

Chr. refers to chromosome, 1 Mbp (Megabase pair) = 1,000,000 bp (base pairs). For the studies of Sun et al. (2020) and Wang et al. (2019), extension and position of the confidence interval around each associated marker were calculated on the basis of LD extension, indicated as ±51 Mbp and 9.6 Mbp, respectively. The physical position of each associated peak marker is as follows: a) BE425919_3_A_592 at 470.072 Mbp; b) IWA2069 at 457.07 Mbp; c) IWA5616 at 457.64 Mbp; d) BE404912_6_B_Y_488 at 352.3 Mbp; and e) BE499652_7_A_Y_391 at 157.5 Mbp.

Clusters 1 and 2 on chromosomes 2A and 2B, respectively, were only found coincident with QTLs for TKW (Patil et al., 2013; Graziani et al., 2014; **Table 6**), whereas the physical interval of cluster 3 related to L and WL overlapped with a QTLs from Desiderio et al. (2019) for the same traits. Regarding cluster 4, it coincided with QTLs for TKW previously detected by Avni et al. (2018) and Sun et al. (2020); meanwhile, the association with this trait was missing in our study. However, two loci described in Wang et al. (2019) in association with kernel W coincided with our result.

Furthermore, the cluster 5 might correspond to a locus associated with TKW detected by Faris et al. (2014) on chromosome 3B, also using a *T. dicoccum–*derived population. However, in the present study, the mentioned QTL was also found related to A, whereas no other traits were previously reported for the same region, which could be an indication of a likely new relationship involving the two traits found here.

The QTL regions associated to PH, kernel W, and TKW in cluster 6 (chromosome 4B) co-localized with five QTLs from previous studies (Patil et al., 2013; Russo et al., 2014; Iannucci et al., 2017; Soriano et al., 2017).

For cluster 7, the region had been previously identified for kernel A and W; however, this was in modern genetic background (Mangini et al., 2021). In addition, QTLs were already found for TKW by Blanco et al. (2012) and Elouafi and Nachit, (2004). In an analogous interspecific durum x emmer population, Russo and co-authors (2014) identified a QTL on chromosome 4B related to TKW and kernel A and W, where the favorable allele was donated from the *T. dicoccum* line. However, this locus was located at about 27 Mbp on the reference genome and thus is unlikely to overlap with our cluster (594 Mbp to 619 Mbp on chromosome 4B). Overall, such comparisons suggested that the cluster 7 detected in this work is likely to be new for the relationships found (between kernel L, P incrementing A, and TKW) as no previous coincidences were found with QTLs for L and P at this specific region.

The physical interval of cluster 8 overlapped with QTLs found related to kernel A, TKW, and WL (Tzarfati et al., 2014; Desiderio et al., 2019; and Sun et al., 2020, respectively).

Only two previous QTLs were found coincident with the physical interval of cluster 9, with one being related to TKW (Patil et al., 2013) and one found in association with kernel W (Sun et al., 2020). This last was consistent with some of the traits associated to this cluster in our work (W, WL, and FC), whereas no coincidences were found for L and P.

Notably, none of our QTLs co-localized with domestication related chromosome regions that are *Q* and *Brt* loci and corresponding cloned genes, respectively, on chromosome 5A and on short arm of chromosome group 3.

### Candidate genes hypotheses for the QTL clusters

Hypotheses about candidate genes, both novel and known cloned genes (**Table 5**), were proposed based on their position within the QTL regions, functional annotations (GO terms), polymorphisms between parent lines (**Appendix G**), and expression profile of bread wheat homologs (**Figure 5**). On the basis of previous knowledge, some GO terms could be more likely associated to functions related to kernel development and grain yield (as hormone pathways and sugar metabolism).

A co-location of yield related traits with QTL for HD, which suggests a pleiotropic relationship, has been described before (Gegas et al., 2010; Mangini et al., 2021). In this study, the physical positions of clusters 1 and 2 (on 2A and 2B, respectively), which also includes QTL for HD, was compared with the known positions of the major genes *Ppd-A1* and *Ppd-B1* (36.6 Mbp and 56.3 Mbp on the Svevo genome, respectively; Maccaferri et al., 2019), which are key components in the photoperiod/flowering regulatory pathway. As depicted in **Table 5**, the physical interval detected in this study for cluster 1 on chromosome 2A included *Ppd-A1*. Instead, cluster 2 on chromosome 2B is slightly shifted in respect to *Ppd-B1*. This effect could be a consequence of the gap present in 2B genetic map or due to the process of anchoring the QTL on the reference genome. Although markers could look to be co-segregant in a genetic map, their physical position on the genome can be slightly different, also based on the recombination rate of the target region. Alternatively, the gene *TRITD2Bv1G019940*, located at 43 Mbp and encoding a coiled-coil domain-containing protein 6G with GO related to controlling cell proliferation, could be a candidate for cluster 2. However, although the bread wheat homolog showed a specific expression in spike at reproductive stages, no SNPs were identified between the MG5323 and Svevo alleles.

Analogously, the pleiotropic consequences of the known *Rht1* (*Rht-B1b*), located at 29.3 Mbp on chromosome 4B, were corroborated with this study, as the semi-dwarfing gene overlapped with the position of cluster 6 (**Table 5**). This gibberellin insensitive dwarfing gene has been comprehensively documented to have pleiotropic effects as an increased grain number and lodging tolerance, which favors grain yield and led to its wide adoption in bread wheat during the Green Revolution. The other pleiotropic effects are reduced seed size, kernel weight, and micronutrient and protein content (Patil et al., 2013; Russo et al., 2014; Mohler et al., 2016; Velu et al., 2017; Guan et al., 2018). The wild-type allele present in emmer makes plants taller and grain larger and heavier as showed by the positive additive effect of the MG5323 allele at cluster 6.

The physical interval of cluster 3 on chromosome 2B did not include any orthologous of known genes related to kernel traits. Noteworthy, in most of the clusters associated with kernel W (clusters 4, 8, and 9), genes known to be involved in/or whose functional annotation is related to cell development were retrieved, strengthening the chances of being potential candidates for this trait. About cluster 4 on chromosome 3A, such type of gene is represented by the wheat orthologous (*TRITD3Av1G163790*) of the known rice gene *D61* (*Os01g0718300*). It encodes a brassinosteroid insensitive–like leucine-rich repeat receptor kinase (Avni et al., 2018) associated with cell elongation (Nakamura et al., 2006). However, the expression of the bread wheat homolog (*TraesCS3A02G245000*) is higher in spikes at vegetative than reproductive stage, and only three SNPs in upstream region were identified between MG5323 and Svevo. Cluster 8 on chromosome 6B encompasses two known genes, *GW2* (*TRITD6Bv1G096950*) and *FUWA* (*TRITD6Bv1G115800*), which are known to control grain size by regulating cell division (Chen et al., 2015; Zhai et al., 2018). Among the two, *GW2* might be the most reliable candidate because the bread wheat homolog (*TraesCS6B02G215300*) showed a specific higher expression in grains at reproductive stage, and three polymorphisms were identified between of MG5323 and Svevo gene sequences. *GW2* encodes an E3 RING ligase and mediates ubiquitination in the ubiquitin–26S proteasome system. This gene has been shown to negatively regulate grain size in rice and in bread wheat (Hong et al., 2014; Simmonds et al., 2016; Nadolska-Orczyk et al., 2017; Zhai et al., 2018). Within cluster 9 on chromosome 7A, annotations of *TRITD7Av1G052720* and *TRITD7Av1G055870*, which encode a receptor protein kinase and a MADS box transcription factor, respectively, mention the regulation of cell growth and cell differentiation. Both these two genes are reliable candidates, because a higher number of SNPs were found between *TRITD7Av1G052720* alleles of MG5323 and Svevo, and a significant upregulation in spike at vegetative stage was seen for the bread wheat homolog of *TRITD7Av1G055870.*

In most of the clusters related to kernel A (clusters 5 and 7), genes associated to auxin metabolism were retrieved *(TRITD3Bv1G229090*, *TRITD3Bv1G229910*, *TRITD3Bv1G235190*, *TRITD3Bv1G239650*, *TRITD4Bv1G175480*, *TRITD4Bv1G179270*, and *TRITD4Bv1G171270*). Several lines of evidence have determined that auxins play an important role in organ size by regulating cell expansion, cell division, and differentiation and thus affecting stem elongation, lateral branching, vascular development, growth responses, and various aspects of seed development, including development of the embryo, endosperm, and seed coat (Teale et al., 2006; Zhao, 2010; Cao et al., 2020b). Among the candidates of cluster 5 on chromosome 3B, two genes (*TRITD3Bv1G229090* and *TRITD3Bv1G235190*) carry polymorphisms in the CDS with moderate effect on the protein. In addition, for the latter, the bread wheat homolog showed a specific induction of gene expression at reproductive stages, including spikes. Specifically in cluster 7, two genes related to auxin regulation (*TRITD4Bv1G175480 and TRITD4Bv1G179270*) and one for cell proliferation (*TRITD4Bv1G177190*) were included. In addition, nearby the physical region of cluster 7, we found the known gene *BIG GRAIN 1* (*BG1*) (corresponding to *TRITD4Bv1G171270* at 582 Mbp), which encodes a plasma membrane–associated protein (Liu et al., 2015) and could be involved in the control of the relationships found for this cluster. This gene overlapped with the position of the loci related to TKW in this cluster (**Figure 4**); therefore, it was considered within the cluster’s interval. In rice, *BG1* (GenBank, accession Q10R09.1) has been described as a positive regulator of the auxin signaling pathway involved in gravitropism, plant growth, and grain development. The overexpressing rice dominant mutants of this gene showed an increased grain size with bigger L, W, and A, associated with longer epidermis cells and higher number of parenchyma cells in both the palea and lemma in the spikelet hull (Liu et al., 2015). Nevertheless, a recent study about the orthologous gene in bread wheat showed that, even if the overexpression of *BG1* led to larger seed size, it also triggered the reduction in seed number per plant (fewer grains), thus causing no significant overall increase in yield, and was related to a lower concentration of essential elements (zinc and phosphorus) and protein content (Milner et al., 2021). Our additional *in silico* evidence could not give a further support to candidate genes of cluster 7. Indeed, none or few polymorphisms were identified a part in *TRITD4Bv1G175480*, which presents three SNPs in upstream gene region, and no specific induction in spike or grain organs, in a general low expression context, was reported for bread wheat homologs. Notably, the bread wheat homolog of the *BG1* gene (*TraesCS4B02G292300*) also showed a very low expression, with a light induction in spike at vegetative stage only.

Within the physical interval of cluster 9 (chromosome 7A), we found a known candidate gene that impacts on grain size by regulating sugar metabolism. This is *TaSus1* (*TRITD7Av1G050690*), which encodes a sucrose synthase, catalyzing the first step in the conversion of sucrose to starch. It has been correlated with TKW, as starch is the main component of grain endosperm (70%) (Nadolska-Orczyk et al., 2017). In addition, within the region of cluster 9, we identified the orthologous gene *TaGASR7* (*TRITD7Av1G071860*), which is considered a negative regulator of grain weight in wheat through an effect on grain L (Dong et al., 2014). Although just a few, both genes have polymorphisms in the upstream gene region.

## Conclusion

Exploring new genetic resources to increase wheat yield is a vital task to cope with climate change and future food demands. In this way, the current study contributes to lay the foundations on understanding the genetic basis on the relationships between kernel-related traits (size, shape, and TKW), by identifying nine clusters of co-located loci in a *T. dicoccum*–derived population. In particular, a major and stable QTL was detected on chromosome 4B related to kernel size traits (L, A, and P) and kernel weight, being the superior allele donated by the *T. dicoccum* accession. This study further supports the role of this ancestral species as a source of favorable alleles for durum wheat breeding although a validation of the detected QTL as with fine mapping is needed to refine the position of the QTL and then study its eventual interaction with other traits and loci.

## Data availability statement

The dataset (variants MG5323-Svevo.v1) presented in this study can be found in the online repository www.figshare.com with the doi: 10.6084/m9.figshare.24119532.

## Author contributions

AV: Data curation, Formal Analysis, Investigation, Writing – original draft. FD: Conceptualization, Formal Analysis, Investigation, Validation, Visualization, Writing – review & editing. RC: Writing – review & editing, Data curation, Formal Analysis. RS: Resources, Writing – review & editing. SR: Writing – review & editing, Resources. AF: Data curation, Software, Writing – review & editing. DG: Data curation, Software, Writing – review & editing. AB: Resources, Writing – review & editing. LC: Conceptualization, Funding acquisition, Resources, Writing – review & editing. EM: Conceptualization, Funding acquisition, Methodology, Resources, Supervision, Writing – review & editing.
