# Supplementary material for: QTL mapping for kernel-related traits in a durum wheat x T. dicoccum segregating population
Source: Front Plant Sci. 2023 Oct 2;14:1253385. doi: 10.3389/fpls.2023.1253385 (PMC10577384; doi:10.3389/fpls.2023.1253385)

**Appendix D.** Pearson correlations coefficients (*r*) among the phenotypic traits analyzed using single environment data: A) Valenzano 2012-2013, B) Bologna 2013-2014, C) Fiorenzuola d’Arda 2014-2015 and D) Fiorenzuola d’Arda 2019-2020. Statistical significance is denoted as *** *p* < 0.001, ** *p* < 0.01 and * *p* < 0.05.


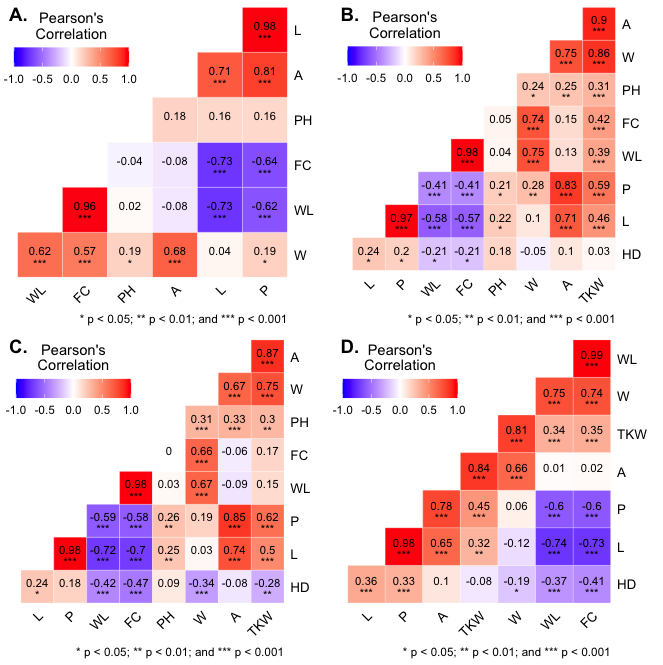

Supplement: Supplementary file 1 [file DataSheet_1.zip › Appendix D.DOCX]
