# Supplementary material for: QTL mapping for kernel-related traits in a durum wheat x T. dicoccum segregating population
Source: Front Plant Sci. 2023 Oct 2;14:1253385. doi: 10.3389/fpls.2023.1253385 (PMC10577384; doi:10.3389/fpls.2023.1253385)

**Appendix F**

1. Sequencing depth across MG5323 chromosomes as computed upon mapping of Illumina paired-end reads of MG5323 against the reference assembly of the durum wheat genome (Svevo.v1, Maccaferri et al., 2019). **
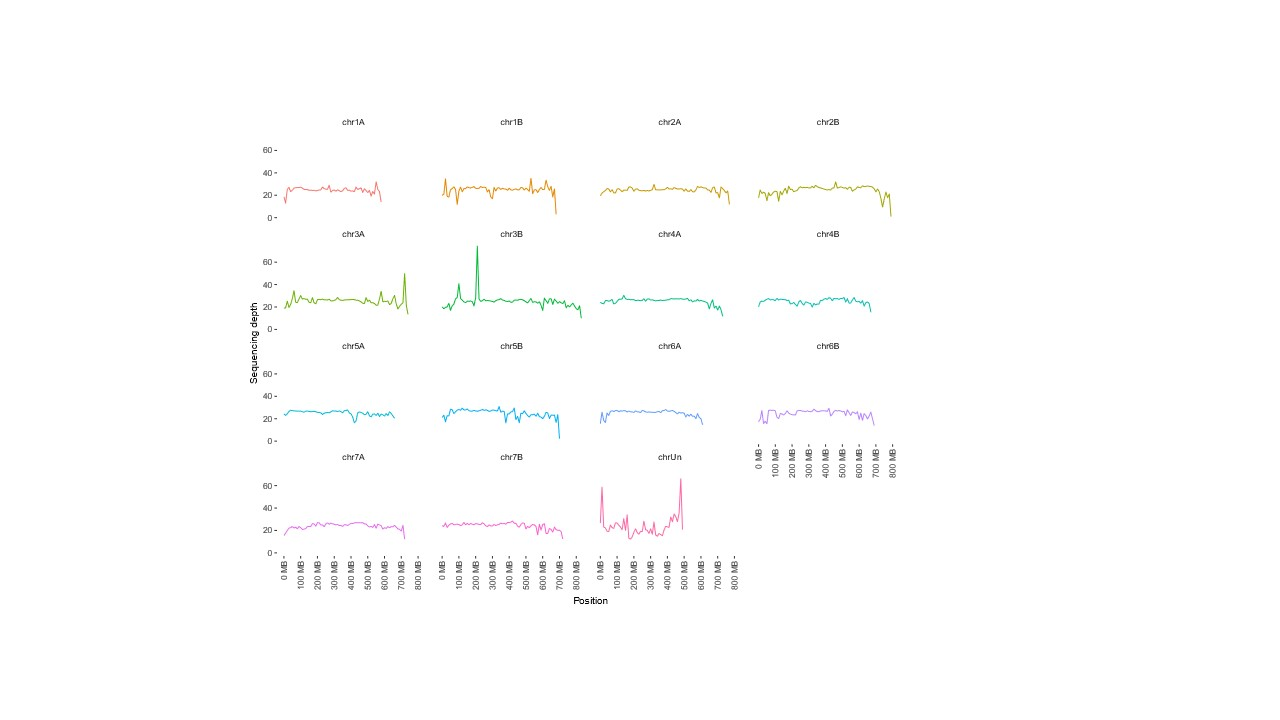
**

**B**) Sequencing coverage computed in 1 Mb windows for the Illumina paired-end whole genome sequencing of MG5323 against the Svevo.v1 assembly.


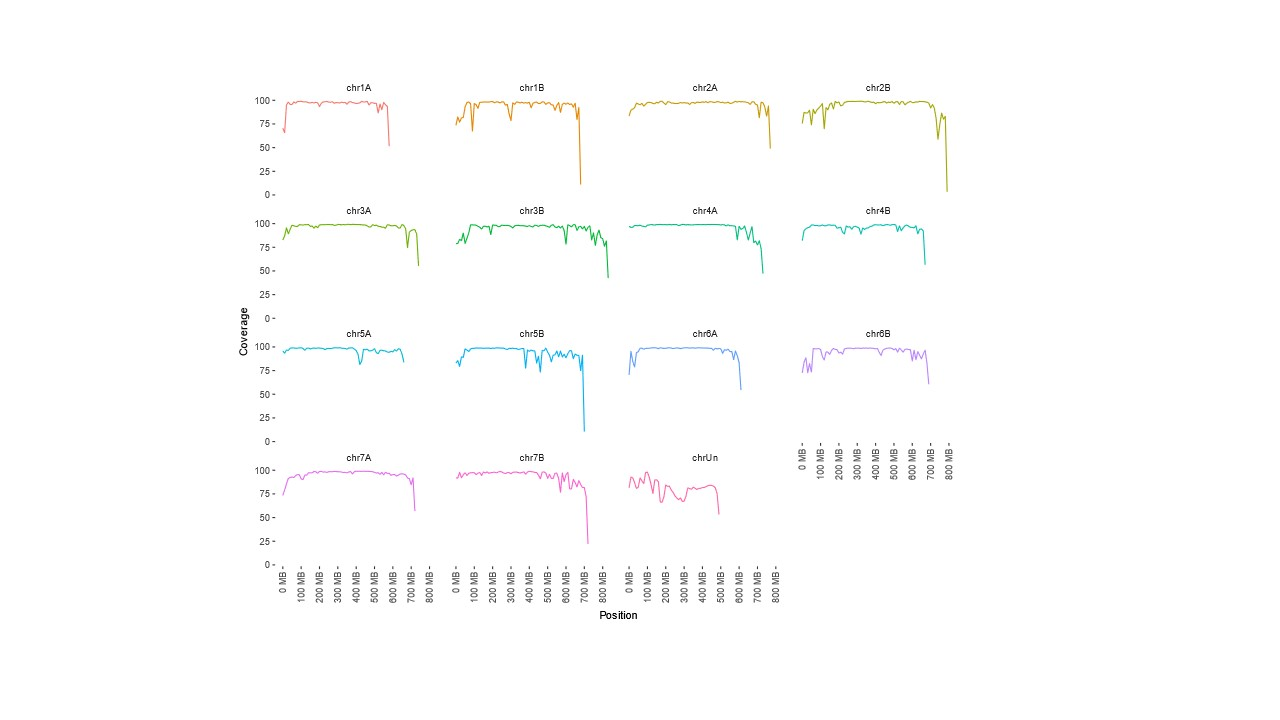

Supplement: Supplementary file 1 [file DataSheet_1.zip › Appendix F.DOCX]
